# Industrial Utilization of Capacitive Deionization Technology for the Removal of Fluoride and Toxic Metal Ions (As^3+/5+^ and Pb^2+^)

**DOI:** 10.1002/gch2.202100129

**Published:** 2022-01-27

**Authors:** Md Rabiul Islam, Soujit Sen Gupta, Sourav Kanti Jana, Thalappil Pradeep

**Affiliations:** ^1^ DST Unit of Nanoscience (DST UNS) and Thematic Unit of Excellence (TUE) Department of Chemistry Indian Institute of Technology Madras Chennai 600 036 India

**Keywords:** arsenic, capacitive deionization, desalination, fluoride, lead, water purification

## Abstract

Capacitive deionization (CDI) is an emerging desalination technology, particularly useful for removing ionic and polarizable species from water. In this context, the desalination performance of fluoride and other toxic species (lead and arsenic) present in brackish water at an industrial scale of a few kilo liters using a CDI prototype built by InnoDI Private Limited is demonstrated. The prototype is highly efficient in removing ionic contaminants from water, including toxic and heavy metal ions. It can remove fluoride ions below the World Health Organization (WHO) limit (1.5 ppm) at an initial concentration of 7 ppm in the input feed water. The fluoride removal efficiency of the electrodes (at a feed concentration of 6 ppm) deteriorates by **≈**4–6% in the presence of bicarbonate and phosphate ions at concentrations of 100 ppm each. The removal efficiency depends on flow rate, initial total dissolved solids, and other co‐ions present in the feed water. Interestingly, toxic species (As^3+/5+^ and Pb^2+^) are also removed efficiently (removal efficiency > 90%) by this technology. The electrodes are characterized extensively before and after adsorption to understand the mechanism of adsorption at the electrode.

## Graphical abstract

The desalination performance of fluoride and other toxic species (lead and arsenic)‐contaminated brackish water using a capacitive deionization prototype built by InnoDI Private Limited is demonstrated. The removal efficiency depends on the flow rate, total dissolved solids, and other ions present in the feed water.

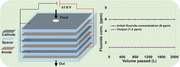

## 1 Introduction

Availability of clean drinking water is one of the major challenges of 21st century. According to the United Nations, more than two‐thirds of the human population will be under water scarcity by 2025.^[^ ^1^, ^2^ ^]^ This is because existing fresh water is getting increasingly contaminated due to a) increased industrialization, b) excessive use of chemical fertilizers in agriculture, c) unprocessed industrial, human, and animal waste, and d) climate change. Groundwater contamination has been gradually increasing with the presence of several toxic contaminants, such as arsenic (As), lead (Pb), fluoride (F^−^), nitrate, and uranium, along with the presence of anthropogenic contaminants such as pesticides, perchlorates, and dyes, thus rendering the water unfit for drinking. Toxic elements such as As and Pb exist in different forms in nature. Exposure to all these contaminants above a specific limit can cause serious health hazards, even carcinogenicity in humans. Among these, fluoride and arsenic are major threats to the Indian population; more than 20 states are severely affected by fluoride and over 12 states by arsenic.^[^ ^3^, ^4^ ^]^ Arsenic contamination in groundwater occurs due to the erosion of natural minerals into aquifers, as a result of complex geochemistry and hydrochemistry.^[^ ^5^, ^6^, ^7^ ^]^ According to the World Health Organization (WHO), over 137 million people in more than 70 countries, and ≈57 million people in 30 countries consume water containing more than 1.5 ppm fluoride and 50 ppb of arsenic, respectively.^[^ ^8^, ^9^, ^10^ ^]^ The standard acceptable limits set by WHO for F^−^ and As are 1.5 ppm and 10 ppb, respectively. Major health effects due to F^−^ intake include dental and skeletal fluorosis. As per reports, excess F^−^ intake can affect the kidney, liver, and reproductive system and cause arthritis, thyroid malfunction, and brain damage.^[^ ^11^ ^]^ On the other hand, long‐term exposure to As through drinking water and food can lead to cancer, cardiovascular diseases, skin lesions, and diabetes.^[^ ^12^ ^]^

In the recent past, researchers have found many efficient solutions for removing As by adsorption using diverse materials.^[^ ^7^, ^13^ ^]^ However, adsorption is not an effective solution to remove F^−^, as its concentration is as high as 2–10 ppm in some parts of the country.^[^ ^14^, ^15^ ^]^ Efforts have been made to remove F^−^ from drinking water using activated alumina, but it is ineffective due to low adsorption capacity and lack of regeneration and reusability without side effects. Activated alumina,^[^ ^16^ ^]^ hybrid graphene oxide (GO)‐ferric hydroxide composite,^[^ ^17^ ^]^ magnetite‐reduced graphene oxide (M‐rGO) composite,^[^ ^18^ ^]^ iron oxide,^[^ ^19^ ^]^ functionalized graphene nanosheets,^[^ ^20^ ^]^ 3D hybrid graphene‐carbon nanotube‐iron oxide composite,^[^ ^21^ ^]^ iron oxyhydroxide‐chitosan composite,^[^ ^22^ ^]^ activated carbon,^[^ ^23^ ^]^ silicon dioxide,^[^ ^24^ ^]^ and cellulose‐based materials^[^ ^6^ ^]^ have been used to remove arsenic from water in the recent past. Among the different heavy metals, Pb has also been widely found in drinking water, which is also highly toxic to human health. Presence of lead, even in trace amounts, can affect nervous, digestive, and skeletal systems, and therefore, must be removed from the water. It has a tendency to accumulate in tissues of living organisms. Major industrial sources of Pb contamination in the environment are battery manufacturing, acid metal plating, and finishing, ammunition, tetraethyl lead manufacturing, ceramic and glass industries, and printing, painting, and dying industries. According to the US Environmental Protection Agency (USEPA), the maximum acceptable level for Pb is 15 ppb (WHO limit: 10 ppb). To achieve this goal, several methods have been applied for the removal of lead from wastewaters, such as precipitation, solvent extraction, ion exchange, coagulation, and floatation, and different materials used for lead removal are fly ash, activated carbon, ion‐exchange resins (IERs), nanosized zero‐valent iron (nZVI)‐based materials, and different graphenic materials.^[^ ^25^, ^26^ ^]^

To meet the demands of safe drinking water globally, several methods were extensively reported for brackish and seawater desalination.^[^ ^27^ ^]^ In the past few decades, desalination techniques including 1) distillation, 2) thermal desalination (multistage flash distillation, multieffect evaporation, vapor compression evaporation, etc.), 3) membrane desalination (reverse osmosis (RO), electrodialysis, membrane distillation, etc.), and 4) ion‐exchange have been developed. However, major disadvantages of these existing techniques are that they are neither cost‐effective nor energy‐efficient. In this context, capacitive deionization (CDI) is emerging as an alternative desalination technology as it is capable of desalination of ionic and polarizable pollutants from brackish water at an affordable cost.^[^ ^28^ ^]^ CDI works on the principle of electroadsorption of ions on porous carbon electrodes when a small potential difference (0.8–2.0 V) is applied across them.^[^ ^29^ ^]^ A CDI cell consists of a pair of porous electrodes (mainly made of carbon), separated by a nonconducting membrane called as separator. When a potential difference is applied across the electrodes, the electrodes get charged, which drives oppositely charged ions toward them by electrostatic attraction. The electrostatic migration continues until an equilibrium is reached, forming an electrical double layer at the interface of the respective electrode. This step is known as electroadsorption, and subsequently, desorption happens when the potential is reversed or the external power supply is shorted. However, the limitation of this technology lies in the availability of sustainable electrode materials with high electroadsorption capacity. The electrode material has a significant role in faster adsorption and desorption kinetics for a perfect CDI process. The electrode material should have the following characteristics: i) large surface area, ii) high porosity, iii) high electrical conductivity, iv) electrochemical stability, v) bio‐inertness, vi) fast adsorption–desorption kinetics, vii) good wetting behavior, viii) low cost, and ix) scalability. Generally, electrode materials used for CDI are mostly carbon‐based materials such as activated carbon, carbon cloth, ordered mesoporous carbon, carbon nanofibers, carbon nanotubes/multiwalled carbon nanotubes (CNTs/MWCNTs), and graphene and graphene‐based composites.^[^ ^30^ ^]^

CDI has numerous advantages. i) It is highly energy‐efficient, as it does not require use of any high‐pressure pumps. ii) The device module works at a lower DC potential ≈0.8–2.0 V. Thus, it can be energized with solar/wind power. More importantly, it can work in rural areas where the availability of grid power is a concern. iii) Water rejection by this technique is significantly less compared to other techniques such as RO. iv) Carbon particles, which are usually used to make active electrodes for CDI, can withstand much higher temperatures than membranes, and therefore can be used for wider applications. Uniqueness of CDI technology over other water purification technologies may be summarized as low operating cost, energy‐efficiency, low wastage, and retention of essential minerals by varying the operating potentials.

In the present work, F^−^ along with other toxic species (As^3+/5+^ and Pb^2+^) containing water was purified efficiently using CDI technology at a scale of relevance for practical applications. This prototype efficiently removed F^−^ below the WHO limit (1.5 ppm) when the concentration of fluoride in feed water was nearly 7 ppm. The same prototype also removed As from 40 to 5.6 ppb and Pb from 200 to 7 ppb, thus bringing output concentrations below acceptable limits. Different experimental conditions, such as flow rate, initial total dissolved solids (TDS), and presence of co‐ions, were optimized to achieve better desalination. Moreover, spectroscopy and microscopy were performed to characterize the electrode surface before and after desalination. The present results show that this CDI prototype is an efficient, cost‐effective, and alternative technology to remove toxic species such as F^−^ along with As^3+/5+^ and Pb^2+^ from contaminated water.

## 2 Result and Discussion

### 2.1 Experimental Set‐Up and Characterization of the Carbon Electrode

**Figure 1:**
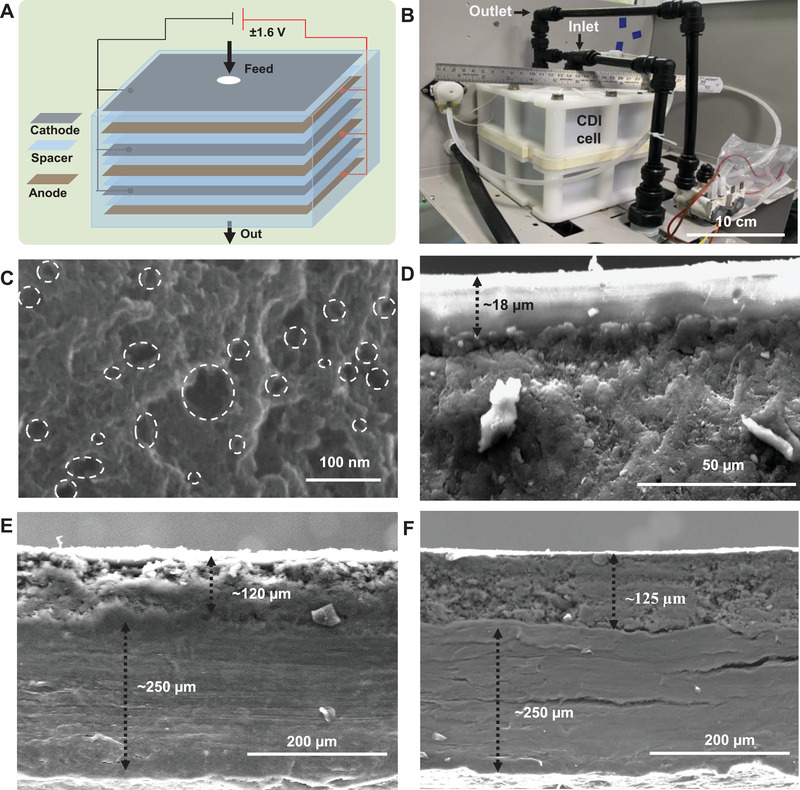
A) Schematic of CDI phenomenon where cathodes and anodes are alternatively stacked, and a DC voltage is applied across the stack B) photograph of the CDI electrode cell. C) HRSEM image of carbon material and HRSEM of the carbon material highlighting the pores present in the nanoscale regime. Cross‐sectional HRSEM images of D) ion‐exchange resins (IERs), E) cathode, and F) anode.

Detailed illustration of the CDI experimental set‐up is provided in Figure S1 in the Supporting Information. The set‐up is composed of several units, each of the units is shown separately. Figure S2 in the Supporting Information shows a photograph of the CDI experimental set‐up. A schematic representation of the CDI phenomenon is demonstrated in **Figure** **1**A, where cathodes and anodes are alternatively stacked, and a DC voltage is applied across the stack. The photograph of CDI electrode cell is shown in Figure 1B. The surface morphology of the electrode material was investigated by high‐resolution scanning electron microscopy (HRSEM), and the corresponding micrograph is shown in Figure 1C. The HRSEM image of the carbon material revealed the highly porous nature of the carbon electrode, which was assembled with porous graphene nanosheets (Figure 1C). We present HRSEM of the electrode materials with 650 000× magnification highlighting the pores present in the electrode materials. From this image, we observed that the electrode materials are highly porous in nature.

Cross‐sectional views of electrodes were also examined through HRSEM images shown in Figure 1D–F. Thickness of the ion‐exchange membrane, which was coated on the electrode material, was found to be ≈15–20 µm (Figure 1D). Additionally, the HRSEM images showed that the thickness of the current collector (i.e., the graphite sheet) of the electrode was around 250 µm, and the graphenic material coated on the current collector had a thickness of about 120 ± 10 µm for both electrodes (Figure 1E,F). The SEM energy‐dispersive X‐ray spectroscopy (EDS) was performed to analyze the distribution of elements in the graphenic carbon electrode. The SEM EDS spectrum of active materials (carbon) of the electrode confirmed the presence of a small amount of oxygen along with carbon, which are the major elements of the electrode material (Figure S3, Supporting Information). The SEM images of the carbon electrodes at different magnifications shown in the inset of the same figure confirm the hierarchical morphology of the active electrodes. The carbon to oxygen ratio was identified as 10.5:1, as per the SEM EDS analysis. The particle size was observed to be 15 ± 5 µm (Figure S3, Supporting Information). Furthermore, surface morphology and elemental mapping of electrodes (cathode and anode) are shown in Figures S4 and S5 in the Supporting Information, respectively. Insets of Figures S4 and S5 in the Supporting Information correspond to EDS mapping of each element. The nanosheets‐like structure of the electrode material masked by an ion‐exchange resin was observed in the SEM image, and smooth surface of the resin was also evident on the electrode surfaces (Figure 1E,F). EDS spectrum of the cathode (Figure S4, Supporting Information) revealed the presence of calcium (Ca), which could be attributed to cation‐exchange resin. Other elements such as carbon and oxygen were also present. However, the presence of Cl^−^ ions at the anodic surface confirms the chemical composition of the anion‐exchange resin (Figure S5, Supporting Information). Figure S6 in the Supporting Information represents the Raman spectrum of the carbon material. The graphenic nature of the material was confirmed by the presence of G‐ and D‐bands at 1604 and 1345 cm^−1^, respectively. Usually, G‐ and D‐bands signify sp^2^ hybridization (graphitic signature of carbon) and disorderness of the sp^2^ hybridized hexagonal sheet of graphenic carbon, respectively. The peak intensity and line‐width of the D‐band are larger than the G‐band in carbon materials, indicating higher disorder/defects, which could be attributed to intense chemical treatments and/or increased amorphous carbon content (unreacted graphite powder). Thus, the Raman spectrum confirms the presence of a graphitic signature of carbon (in‐plane sp^2^ carbon) and defects present in carbon particles (sp^3^ carbon).

### 2.2 Electrochemical Characterization of both Anode and Cathode Materials

Cyclic voltammetry (CV) was carried out in 1 m NaCl solution to understand the adsorption–desorption and capacitive behavior of the electrodes (anode and cathode). Voltammograms of both electrodes at different scan rates, from 1 to 100 mV s^−1^, are shown in Figure S7A,B in the Supporting Information. The voltammograms revealed that both anode and cathode are perfectly reversible at lower scan rates. These attributes to anodic and cathodic currents (or capacitive currents) are the mirror images, indicating that both adsorption and desorption processes occur almost at the same kinetic rate. However, at higher scan rates (beyond 50 mV s^−1^), alteration in the shape of the CV profile of each electrode was monitored, implying that less amount of ions was adsorbed at electrodes. This result suggests that ions do not have enough time to access the entire electrochemical surface of the electrode material. Thus, at higher scan rates, adsorption and desorption of ions are limited by ionic resistance. Specific capacitance (*C*_sp_) of both the electrodes at each scan rate was calculated and plotted as a function of scan rate (Figure S7C,D, Supporting Information). Exponential decay of the specific capacitance exhibits a higher value (≈68 F g^−1^) of *C*_sp_ at a lower scan rate and gets constant at a higher scan rate. This could be explained as the adsorption and desorption are faster at a lower scan rate as ions have enough time to get adsorbed on the oppositely charged electrode surfaces. This phenomenon is similar to the charge storage mechanism of an electrochemical capacitor. However, at a higher scan rate, the diffusion‐controlled process dominates; therefore, adsorption and desorption rates are lower. Also, high salt adsorption capacity of the electrode material was observed, as seen in the electrochemical study (Figure S7, Supporting Information), which further confirms the porous nature of the electrode materials.

Scan rates‐dependent adsorption–desorption characteristics were studied for anode and cathode in 1 m of NaCl and NaF solutions. At a lower scan rate (1 mV s^−1^), the charge storage capacities (area under the curve of the voltammograms) of both the electrodes were found to be the same (Figure S8A, Supporting Information). However, at a higher scan rate (100 mV s^−1^), CV of both anode and cathode was performed in 1 m NaCl solution, and the corresponding voltammogram showed the difference in charge storage capacities (Figure S8B, Supporting Information). As the ionic mobility of Na^+^ and Cl^−^ are different (5.19 and 7.92 m^2^ s^−1^ V^−1^, respectively), and diffusion of ions is controlled by the ionic mobilities of the counter‐ions (cations for cathode or anions for anode). Therefore, the cathode shows a significantly higher charge storage capacity than the anode. The same experiment was carried out in 1 m NaF solution, which does not show any significant change in the charge storage capacity. However, ionic mobilities of both Na^+^ and F^−^ are almost the same (5.19 and 5.74 m^2^ s^−1^ V^−1^, respectively). Thus, CV of both the electrodes, which were performed in 1 m NaF solution, showed the same charge storage capacity at the same scan rate. Interestingly, at a lower and higher scan rate (1 and 100 mV s^−1^, respectively), voltammograms of both the electrodes are almost similar (Figure S8C,D, Supporting Information). This indicates that the capacitive currents of the electrodes are equal for both scan rates.

In order to understand charge transport at the electrode–electrolyte interface and its effect on capacitive desalination, electrochemical impedance spectroscopy (EIS) measurement was carried out for both anode and cathode. EIS was performed in 1 m NaCl solution by applying 10 mV sinusoidal AC signal to the working electrode (here, graphite electrode coated with carbon material), and the frequency of the input signal was varied from 5 MHz to 1 mHz. The total impedance of the electrochemical cell was recorded at 10 dB per decade of the applied frequency, and the Nyquist plots of both cathode and anode are shown in Figure S9A,B in the Supporting Information. The Nyquist plot represents the impedance of the working electrode at each frequency. An equivalent circuit was deduced by fitting the impedance data with the experimental Nyquist profile of the individual electrode. Fitting vales of an equivalent circuit of both cathode and anode are shown in Table S1 in the Supporting Information. Each circuit element, which is discussed in the caption of Figure S9C in the Supporting Information, is analogous to different interfacial electrochemical phenomena. These phenomena are involved with i) diffusion of bulk ions to the electrode–electrolyte interface, ii) charge transfer through adsorption and desorption of ions at the electrode surface (*R*_ct_), and iii) charge transport through the active carbon material to the graphite electrode (current collector, R1). Analysis of Nyquist plots and corresponding equivalent circuits revealed internal resistance of the anode (≈38.3 Ω) to be higher than the cathode (≈12.5 Ω). This signifies that the electronic conductivity of the cathode material is higher than the anode material. Therefore, at the same scan rate, the capacitive current is lower for the anode compared to the cathode (Figure S8, Supporting Information). However, charge transfer resistance is almost the same for the two electrodes (for cathode ≈7.1 Ω and anode 9.8 Ω), which is attributed to the same adsorption and desorption rate of ions on the electrodes. CDI works on the principle of physical adsorption/desorption of ions. However, there was no significant difference in adsorption (charge) and desorption (discharge) rates. Even after 3 h of continuous adsorption and desorption cycles, electrode surface was usually regenerated in CDI technology to recover the active sites of the electrode for further adsorption and desorption processes. However, there is a difference in diffusion impedance [*Z*_D_ = C3║R3] between anode and cathode, i.e., R3 of the anode is three times higher than the cathode, attributed to the slightly lower adsorption rate at the surface of the anode (Table S1, Supporting Information).

### 2.3 Adsorption–Desorption Experiment of the Electrode

**Figure 2:**
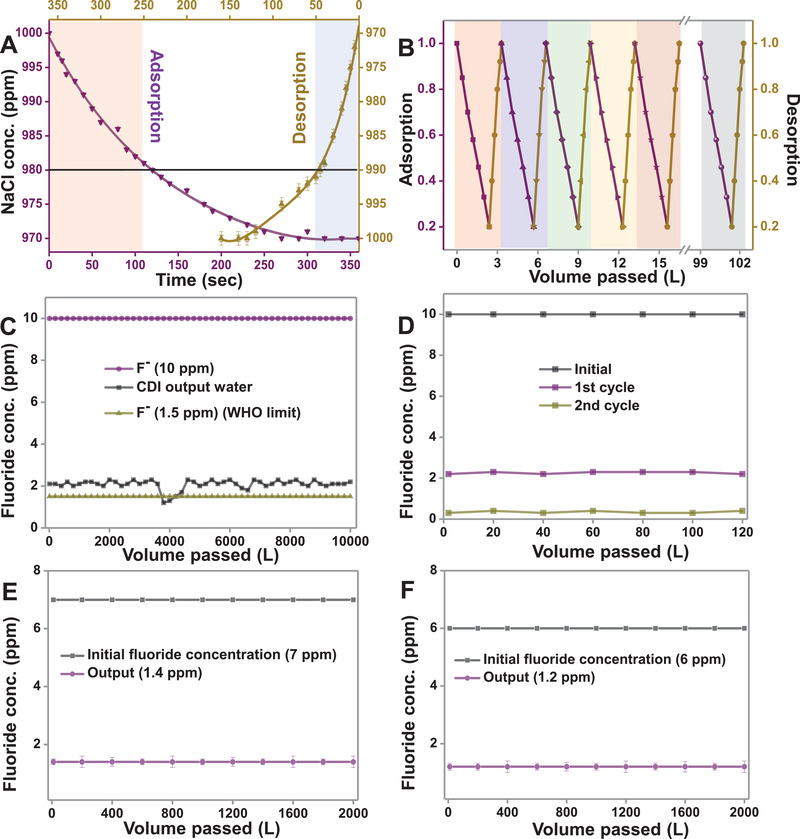
A) Single adsorption–desorption cycle containing 1000 ppm of NaCl in batch mode and B) adsorption–desorption performance in a continuous flow‐through mode for multiple cycles, CDI performance for the removal of fluoride ion in tap water with initial concentration C) 10 ppm for 10 000 L, D) 10 ppm for a double pass for 120 L, E) 7 ppm for 2000 L, and F) 6 ppm for 2000 L.

Adsorption–desorption measurement was performed in batch mode with single electrode pair (cathode and anode, each of the dimensions 3 × 5 cm^2^) immersed in 1000 ppm of 80 mL NaCl solution and a DC potential of ±1.6 V was applied across them. After 360 s, a decrease in the concentration of the solution to 970 ppm during the adsorption cycle was noticed. After reversing the terminal, complete desorption was noticed at 200 s to reach the initial concentration (1000 ppm), as shown in **Figure** **2**A.

To get insights into the adsorption and desorption kinetics, both adsorption and desorption profiles were analyzed at different time segments. A similar experiment was reported in our previous work.^[^ ^31^ ^]^ In this work, fast adsorption kinetics was observed for 20 ppm in the first 125 s, while desorption of the same concentration of ions was seen in 45 s. Therefore, in flow‐through experiments (when NaCl solution was passed through the cell), the time of adsorption/desorption was kept at 120/45 s to achieve maximum desalination efficiency. Figure 2B shows the efficiency of CDI electrodes in a flow‐through experiment for multiple cycles (for 30 cycles). The initial concentration was kept at 1000 ppm, and the output concentration was found to be 200 ppm after 120 s of adsorption. After desorption, the concentration in the rejected water was measured to be 3140 ppm after 45 s at a flow rate of 120 L h^−1^.

Different experiments were carried out to evaluate the CDI performance for the removal of F^−^ from input feed water. Different concentrations of F^−^ were spiked in tap water, and the removal efficiency was measured. The initial TDS of tap water was ≈950 ppm, and the quantitative analysis of ions was measured by ion chromatography (IC). When the initial concentration of F^−^ was 10 ppm, the output concentration of the same was found to be 2–2.1 ppm, after passing 10 000 L of water through the electrochemical cell during capacitive desalination (Figure 2C). The reduction of TDS was around 80%, i.e., from ≈1000 to ≈200 ppm. However, a sudden decrease in fluoride concentration below 2 ppm was witnessed (black trace of Figure 2C) when 4000 L of tap water was passed during the experiment. This fluctuation might be because of lower TDS of input tap water below a certain value. The output water having F^−^ concentration of 2–2.1 ppm was then passed through the CDI cell for the second cycle (i.e., double pass), and the concentration of F^−^ reduced to 0.4 ppm, which is below the acceptable limit in drinking water as per WHO's standard (Figure 2D). Therefore, for a higher concentration of F^−^, a double pass is required to bring the F^−^ concentration below 1.5 ppm. However, the removal efficiency depends on the flow rate, initial TDS, and the presence of co‐ions.

The typical concentration of F^−^ in groundwater and surface water bodies of fluoride‐affected areas in India was reported to be 0.5–6 ppm (**Table** **1**). Few places had fluoride ion concentrations higher than 10 ppm, as in Prakasham district in Andhra Pradesh, Unnao district in Uttar Pradesh, and Karbianglong district in Assam. When F^−^ concentrations of 7 and 6 ppm were spiked into the input tap water, output concentrations of F^−^ were observed to be 1.4 and 1.2 ppm, respectively (Figure 2E,F). The observed result suggests that the CDI module could efficiently remove F^−^ (below WHO limit) from water with an input F^−^ concentration of ≈7 ppm or lower. For higher concentrations (above 7 ppm), a double pass methodology is required. Additionally, CDI performance using different concentrations of F^−^ was also studied, in which the initial concentration of the F^−^ was maintained as 100, 50, 10 ppm (Figure S10A–C, Supporting Information). Moreover, the effect of different TDS and flow rates were also studied with an input concentration of 10 ppm of F^−^ (Figure S10D, Supporting Information).

**Table 1:** Typical concentration of fluoride ions found at fluoride‐affected areas in India

S. no.	First author(ref.)	Year	State/location	Fluoride conc. [ppm])
1	N. Subba Rao^[^ ^32^ ^]^	2003	Andhra Pradesh/Guntur	0.60–2.30
2	N. Subba Rao^[^ ^33^ ^]^	2003	Andhra Pradesh/Anantapur	0.56–5.80
3	P. D. Sreedevi^[^ ^34^ ^]^	2006	Andhra Pradesh	0.38–4.00
4	D. Sujatha^[^ ^35^ ^]^	2003	Andhra Pradesh/Ranga Reddy	0.40–4.80
5	J. Dutta^[^ ^36^ ^]^	2010	Assam/Sonitpur	0.17–5.60
6	N. J. Raju^[^ ^37^ ^]^	2009	Uttar Pradesh/Sonbhadra	0.48–6.70
7	P. Sharma^[^ ^38^ ^]^	2012	Assam/Nalbari	0.02–1.56
8	S. Ramanaiah^[^ ^39^ ^]^	2006	Andhra Pradesh/Prakasam	0.50–9.0
9	S. Gupta^[^ ^40^ ^]^	2006	West Bengal/Birbhum	0.01–1.95
10	Meenakshi^[^ ^41^ ^]^	2004	Haryana/Jind	0.3–6.9
11	G. Karthikeyan^[^ ^42^ ^]^	2000	Tamil Nadu/Dharmapuri	0.98–5.60
12	P. Kotecha^[^ ^43^ ^]^	2012	Gujarat/Vadodara	0.02–4.17
13	N. Sankararamakrishnan^[^ ^44^ ^]^	2008	Uttar Pradesh/Kanpur	>1.5
14	C. R. Ramakrishnaiah^[^ ^45^ ^]^	2009	Karnataka/Tumkur	0.02–3.2
15	A. Shivashankara^[^ ^46^ ^]^	2000	Karnataka/Gulbarga	0.6–13.4
16	G. Viswanathan^[^ ^47^ ^]^	2010	Tamil Nadu/Dindigul	0.76–3.17
17	M. Bishnoi^[^ ^48^ ^]^	2007	Haryana/Rohtak	0.03–2.09
18	D. V. Reddy^[^ ^49^ ^]^	2010	Telangana/Nalgonda	0.50–7.00
19	D. R. R. Sarma^[^ ^50^ ^]^	1997	Andhra Pradesh/Visakhapatnam	0.24–8.10
20	S. K. Jha^[^ ^51^ ^]^	2010	Uttar Pradesh/Unnao	0.8–13.9
21	M. Arif^[^ ^52^ ^]^	2012	Rajasthan/Nagaur	0.4–6.6
22	S. Yadav^[^ ^53^ ^]^	2011	Uttar Pradesh/Rampur	0.32–1.80
23	P. Kotoky^[^ ^54^ ^]^	2010	Assam/Karbi Anglong	0.95–20.60
24	A. C. Samal^[^ ^55^ ^]^	2015	West Bengal/Purulia	0.01–1.6
25	H. Pauwels^[^ ^56^ ^]^	2015	Andhra Pradesh/Maheshwaram	0.26–3.73
26	K. S. Patel^[^ ^57^ ^]^	2017	Chhattisgarh/Rajnandgaon	3.7–27
27	S. Manikandan^[^ ^58^ ^]^	2014	Tamil Nadu/Krishnagiri	0.50–5.45

**Figure 3:**
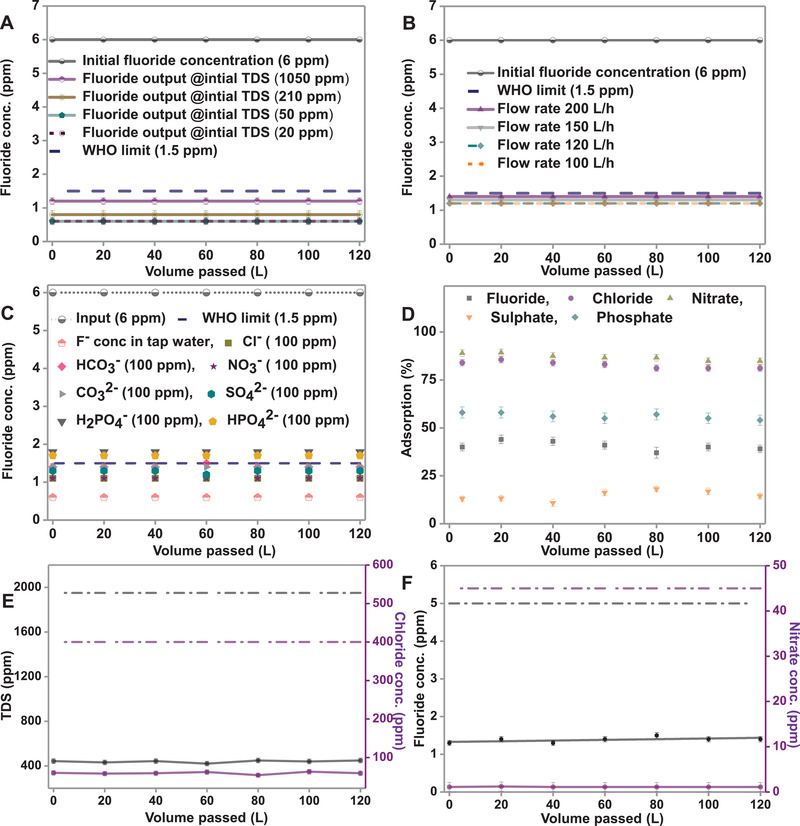
CDI performance for removing F^−^ at an initial concentration of 6 ppm at A) different initial TDS and B) different flow rates, C) different co‐ions spiked in tap water, and D) adsorption efficiency for anions in a mixture. Effect of E) TDS and Cl^−^, and F) F^−^ and NO_3_^−^ were studied on the CDI performance.

Effect of removal efficiency of F^−^ at an input concentration of 6 ppm was also examined with variation in TDS and flow rate. Initially, TDS of tap water was measured to be 1050 ppm, which, upon passing through the CDI module, reduced to 210 ppm in the output water. Similarly, when 210 and 50 ppm of water were passed through the CDI module, the output water was at 50 and 20 ppm, respectively. Water samples of different TDS, each of 120 L volume, were collected from the IITM tap water, and further, F^−^ was spiked in each water sample in such a way that the final concentration of F^−^ was maintained at 6 ppm. After performing desalination through CDI, the output concentrations of fluoride were found to be 1.2, 0.8, 0.5 ppm for input TDS of 1050, 210, and 50 ppm, respectively (**Figure** **3**A). Even when the TDS of input water was reduced to below 50 ppm, and the F^−^ concentration was kept fixed at 6 ppm, the output F^−^ concentration was found to be 0.5 ppm. The flow rate was kept fixed at 120 L h^−1^ for the above experiments.

F^−^ removal efficiency was also checked as a function of flow rate (Figure 3B). With input concentrations of F^−^ and TDS at 6 and 1050 ppm, respectively, the output concentrations of F^−^ were observed as 1.4, 1.3, and 1.2 ppm with flow rates of 200, 150, and 120 L h^−1^, respectively. The limit of removal efficiency is attributed to the constraint of flow rate, as the ions do not have enough time to get adsorbed onto the electrodes. The fluoride removal efficiency of CDI in the presence of co‐ions was also investigated. At a constant concentration of 6 ppm of F^−^, CDI experiments were performed in the presence of Cl^−^, NO_3_^−^, HPO_4_^2−^_,_ H_2_PO_4_^−^_,_ SO_4_^2^, HCO_3_^−^_,_ and CO_3_^2−^, each at 100 ppm. The removal efficiency of F^−^ exhibited a negligible effect for 100 ppm Cl^−^, NO_3_^−^, or SO_4_^2−^ (Figure 3C). However, the adsorption efficiency of the electrode decreased drastically when phosphates (HPO_4_^2−^ and H_2_PO_4_^−^) or bicarbonate (HCO_3_^−^) were added to the input solution (Figure 3C).

Selectivity and the removal efficiency of F^−^ were also studied in the presence of a mixture of Cl^−^, NO_3_^−^, SO_4_^2−^, F^−^, and PO_4_^3−^, each at 100 ppm (Figure 3D). The data suggested that ion adsorption kinetics follows the following order: NO_3∼_ ≥ Cl^−^> PO_4_^3−^ > F^−^ > SO_4_^2−^. Figure 3E,F demonstrates the effect of TDS, Cl^−^, and NO_3_^−^ on the adsorption of F^−^. When synthetic water (TDS maintained at ≈1957) containing 5 ppm of F^−^, along with 400 ppm of Cl^−^ and 45 ppm of NO_3_^−^, was passed through the CDI system, the output concentration of F^−^ was 1.2 ppm, and a significant reduction in TDS (from ≈1957 to ≈415 ppm) was also observed (after one cycle). Our results show that F^−^‐contaminated real water could be purified efficiently using this technique.

**Figure 4:**
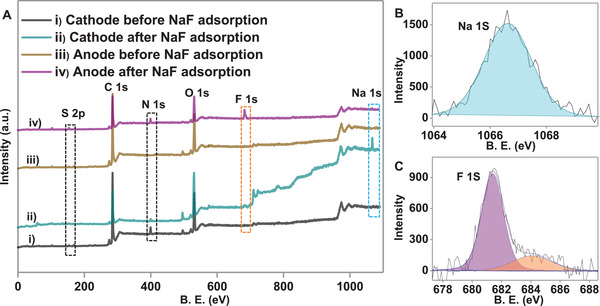
A) XPS survey spectra of the material after single‐stage adsorption on i) cathode and iii) anode (before adsorption); ii) cathode and iv) anode (after NaF adsorption), B) and C) are the deconvoluted XPS spectra of Na 1s (light blue) and F 1s (pink@681.4 eV and orange color@684.5 eV) for cathode and anode, respectively after NaF adsorption.

Post adsorption, electrodes were characterized thoroughly using both SEM EDS and X‐ray photoelectron spectroscopy (XPS) to confirm the adsorption of F^−^. Figure S11 in the Supporting Information shows the SEM EDS spectrum of the electrodes after the adsorption of NaF onto them. Figure S11A in the Supporting Information shows that cations (Na^+^ ions) were adsorbed on the cathode, confirmed by elemental mapping (shown in the inset). Similarly, Figure S11B in the Supporting Information confirmed that counter ions (F^−^ ions) were adsorbed on the anode surface. **Figure** **4**A shows the XPS survey spectra of both anode and cathode before and after the adsorption of NaF solution. In the XPS survey spectra, no significant changes were observed in the binding energies of carbon, nitrogen, oxygen, and sulfur of both cathode and anode, even after electrochemical adsorption, which justifies that adsorption is physical in nature. The XPS survey spectra (Figure 4A) and deconvoluted XPS spectra (Figure 4B,C) revealed the adsorption of Na^+^ and F^−^ ions at cathode and anode, respectively. The XPS data also suggest that only Na^+^ and F^−^ ions were absorbed on cathode and anode, respectively. Moreover, deconvoluted XPS spectra of C 1s at both electrodes before and after NaF adsorption revealed no significant changes in binding energy, which validates that electroadsorption is physical in nature (Figure S12, Supporting Information).

**Figure 5:**
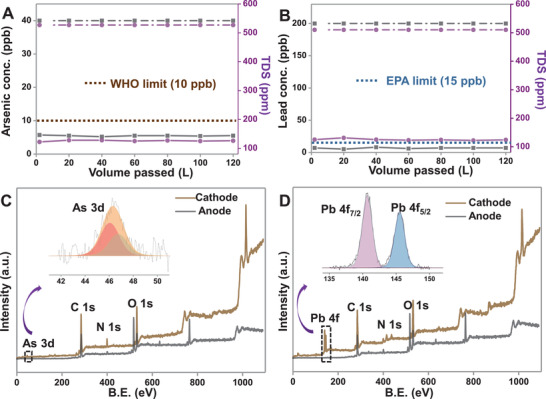
CDI performance for the removal of toxic ions (arsenic and lead) in tap water with initial concentration: A) 40 ppb of As (as As^3+/5+^) and B) 200 ppb of Pb (as Pb^2+^); XPS survey spectra of the material after single‐stage adsorption on cathode and anode C) after arsenic adsorption, and D) after lead adsorption. Deconvoluted XPS spectra of As 3d Pb 4f regions are shown in the inset.

**Figure** **5**A,B shows that CDI can efficiently remove other common toxic metal ions such as As^3+/5+^ and Pb^2+^ from water. To investigate the desalination performance of CDI for As^3+/5+^ removal, the input concentration of As was maintained at 40 ppb (As^+3^:As^+5^ = 1:1), and it was spiked into tap water. The concentration of As in the permeate water (production water) was reduced to ≈5.6 ppb when the spiked water was run through the CDI unit, which is below the acceptable limit set by WHO (Figure 5A). Thus, permeate water after desalination is fit for drinking purposes. Moreover, 200 ppb of Pb^+2^ was also spiked into the tap water and was run through the CDI cell; the permeate water concentration of ≈7 ppb was obtained, which is below the Environmental Protection Agency (EPA) and WHO limits (Figure 5B). To check the repeatability, the adsorption experiments for Pb and As were performed five times (≈1100 L of water was tested for both ions). The detailed adsorption mechanisms are shown in Figures S13–S16 in the Supporting Information. SEM EDS data confirmed that only cations were adsorbed on the cathode and anions on the anode. Figure 5C,D displays the XPS survey spectra of the cathode after adsorption of As and Pb, which revealed that both As and Pb could be removed. Insets of both Figure 5C,D show deconvoluted spectra of As and Pb on the cathode. XPS data confirm that cations are removed at the cathode and anions at the anode. XPS data also indicate that ions are almost completely removed from both the electrodes, and adsorption sites at the electrode surfaces are regenerated.

## 3 Conclusion

The present work demonstrates the desalination performance of a CDI prototype against brackish water containing F^−^ along with other toxic species (As^3+/5+^ and Pb^2+^), at an industrial scale. The CDI technology efficiently removes F^−^ concentration below the WHO limit when the concentration of fluoride in feed water is ≈7 ppm. For higher input F^−^ concentration, a double pass is required to bring the F^−^ concentration below the prescribed limit. The fluoride removal efficiency of the electrodes depends on flow rate, initial TDS, and co‐ions present in the water. Moreover, the removal efficiency is reduced in the presence of phosphates and bicarbonates in the water. Additionally, the CDI prototype was utilized to remove other toxic species such as As^3+/5+^ and Pb^2+^ from contaminated water. The electrodes were characterized extensively before and after adsorption to check the adsorption mechanism, which revealed that the process (adsorption/desorption) is physical in nature. Electrodes also exhibited high electroadsorption performance, fast deionization rate, and good regeneration capability. The CDI technology could thus be an efficient and alternate way to remove toxic ions from brackish water.

## 4 Experimental Section

### Materials

Sodium chloride (NaCl) and glacial acetic acid were purchased from RANKEM, India. Sodium hydroxide (NaOH) was purchased from Merck. Sodium dihydrogen ortho‐phosphate (NaH_2_PO_4_), disodium phosphate (Na_2_HPO_4_), and sodium fluoride (NaF) were purchased from Fisher Scientific, India. Sodium sulfate (Na_2_SO_4_), sodium nitrate (NaNO_3_), sodium nitrite (NaNO_2_), sodium carbonate (Na_2_CO_3_), sodium bicarbonate (NaHCO_3_), and potassium chloride (KCl) were purchased from RANKEM, India. All chemicals were of analytical grade and were used as received without further purification.

### Instrumentation

SEM equipped with EDAX (FEI Quanta 200, Czechoslovakia) was used to record the surface morphology, elemental composition, and elemental mapping of the samples. Also, HRSEM images of the electrode materials were obtained through Thermo Scientific Verios G4 UC SEM. XPS measurements were done with an Omicron ESCA Probe spectrometer with polychromatic Mg Kα X‐rays. Most of the spectra were deconvoluted to their component peaks using the Casa XPS software. The energy resolution of the spectrometer was set at 0.1 eV at pass energy of 20 eV. Binding energy was corrected with respect to C 1s at 284.5 eV. Raman spectra were obtained with a WITec GmbH, Alpha‐SNOM alpha 300 S confocal Raman microscope having a 532 nm laser as the excitation source. The Eutech Cyber scan PC650 multiparameter monitor supplied by Thermo Scientific, India, was used for measuring conductivity, pH, and fluoride concentration. Ion‐exchange chromatography or Ion chromatography (883 Basic IC plus model) was used for quantitative analysis of common anions (such as fluoride, chloride, nitrate, nitrite, and sulfate) in an aqueous solution. Metrosep A Supp 5 – 250/4.0 column (Order number: 6.1006.530) was used, and polyvinyl alcohol with quaternary ammonium groups was used as carrier materials for anion detection. Sodium carbonate (3.2 × 10^−3^ m) and sodium bicarbonate (1 × 10^−3^ m) mixture were used in 1:1 ratio for anion sample detection. In addition, 100 × 10^−3^ m H_2_SO_4_ and deionized water (DI) were used as suppressor eluents for cleaning the column.

The electrochemical capacitive behavior of carbon electrodes was determined by CV using a CH Electrochemical Analyzer (CH 600A). The CV was performed at various scan rates (1–100 mV s^−1^) in a potential range of −1.0 to +1.0 V. The specific capacitance was calculated from the CV curve based on the following equation

(1) $$\begin{array}{c} C_{s}=\left(\frac{1}{mR\Delta V}\right)\;\times \int I\left(V\right)dV \end{array}$$

where *C*_s_ is the specific capacitance, *m* is the mass of the active material, *R* is the scan rate, d*V* is the potential window of scanning and ∫I(V)dV is the integral area under the CV curve. The electrochemical capacitive behavior of the electrode materials was determined by CV.

All electrochemical experiments were carried out at room temperature using 1 m NaCl and 1 m NaF electrolytes solution in a three‐electrode cell adopted with a carbon electrode as the working electrode, a platinum electrode as the counter electrode, and Ag/AgCl (3 m KCl) as the reference electrode. The Eutech Cyber scan PC650 multiparameter monitor supplied by Thermo Scientific, India, was used for ionic conductivity and fluoride ion measurement.

### Fabrication of CDI Electrodes

Normally polyvinyl fluoride (PVDF) and polyvinyl alcohol (PVA) are used as binders for CDI electrodes. Here, for preparation of anode and cathode materials, PVDF and carbon material were taken in different ratios in dimethylformamide, and different optimizations were done. Best electrode material was obtained after wet grinding for 4 h (using a normal grinder). The optimized electrode slurry was coated on both sides of graphite sheets (thickness ≈350 µm). Both coating was performed by doctor blade technique and curing of the coated electrode was done at 120 °C for 1.5 h. The coating thickness of carbon materials was about ≈125 µm in both sides (Figure 1E,F). Further, optimized electrodes were coated with ion exchange resins (IERs) using a spray gun and dried with an IR heater at 340 °C for 1–2 min. Dried electrodes were kept under water until further use (Figure 1D). Finally, the dimension of the CDI electrodes was maintained as ≈10 × 10 cm^2^ for desalination purpose. The CDI module was designed with an assembly of hundred pairs of electrodes.

It was known that thickness of the electrode materials would play an important role in the performance of the cell. It was well known that the adsorption capacity of the electrode would be increased with increasing the material loading on the substrate. But, the optimized thickness of the active electrode material (both carbon material and ion‐exchange resins (IERs)) on the substrate was the utmost criterion for the CDI electrode design. Moreover, the physical stability of the electrode would be lost with higher loading of the electrode material, and eventually, electrodes would also lose their adsorption efficiency. Therefore, the optimum thickness of the electrode material was taken care of for higher removal efficiency. In this case, the optimum thickness of ≈120–140 µm of carbon material coating with IERs thickness of 20–30 µm was maintained, on top of the active material. It was shown (Figure 2C) that there was no change of adsorption capacity observed even after passing 10 000 L of water continuously through the CDI cell. Therefore, it was concluded that this electrode material with optimum thickness was stable for the process of desalination.

### Fluoride Removal Experimental Set‐Up for CDI

CDI unit was built, as shown in the schematic in the Supporting Information (Figure S1, Supporting Information). A 0.5 HP pump was used in the unit. The flow rate of water was controlled by a valve from 100 to 200 L h^−1^. The unit consisted of three pre‐filtration stages, a 50 μm cartridge for unsuspended particles, a carbon filter for organics, color, and odor, and 10 μm cartage for any smaller unsuspended particle. Before performing the desalination study using CDI cells, the raw water (DI) was passed through a UV chamber for bacterial remediation. The CDI cell consisted of 100 pairs of electrodes (each of the dimensions 10 × 10 cm^2^), and water was passed in flow‐through mode. An interelectrode spacing of ≈0.2 mm was maintained with a nylon membrane for the electrode cell assembly. The electrochemical performance of the CDI cell was automated by electronic circuitry such that the adsorption cycle lasted for 120 s, and the desorption cycle for 45 s. The cells were connected to a DC power source, with voltage ranging from 0.8 to 2 V. The flow rate for all adsorption and desorption cycles was maintained at 120 L h^−1^.

The CDI modules generally were expressed as electrode pairs; if the number of CDI electrode pairs were increased, the adsorption efficiency would be increased. Consequently, the removal efficiency would be increased. In the CDI module with 100 electrode pairs, ion adsorption capacity or salt removal capacity was 80% with an input TDS of 1000–3000 ppm of NaCl solution. The production capacity of the CDI module was 2000 L per day (LPD). It was seen that maximum water rejection in this prototype was ≈18% for all the contaminants.

CDI was explored extensively for brackish water desalination under the premise of being energetically competitive with RO. It was seen that quanta of energy consumed by different CDI and RO were comparable in identical conditions. However, the energy expenditure of experiments was not reported which were carried out using membrane capacitive deionization (MCDI) prototype. However, it was reported that the energy requirement of MCDI was two to three times higher than RO for brackish water desalination (using the same condition such as water recovery (WR) = 80%, salt rejection (*R*_salt_) = 80%, flux (*J*_w_) = 10.0 L m^−2^ h^−1^, and feed salinity of 34.22 × 10^−3^ m (2 g L^−1^)).^[^ ^59^ ^]^ It was also shown that in the same condition, energy consumption of MCDI (≈0.4 kWh m^−3^) was less than CDI (≈2.5 kWh m^−3^).^[^ ^59^ ^]^ Furthermore, it was seen that for these desalination conditions (water recovery (WR) = 93.5%, salt rejection (*R*_salt_) = 80%, flux (*J*_w_) = 11.9 L m^−2^ h^−1^, and a feed salinity of 40 × 10^−3^ m), the energy consumption of RO (≈0.5 kWh m^−3^) was higher than of MCDI (≈0.4 kWh m^−3^).^[^ ^59^ ^]^ As similar desalination conditions were maintained for brackish water using this prototype, it was believed that energy consumption of this prototype was similar during desalination. The cost of desalination was also calculated using this CDI prototype, which was ≈3–4 paisa (US$0.00040 to 0.00054) per liter.

### TISAB (Total Ionic Strength Adjustment Buffer) II Preparation

First, 500 mL DI water was taken in 1 L beaker, and 57 mL of glacial acidic acid was added to it; the solution mixture was then allowed to stir for 10 min. Subsequently, 58 g of sodium chloride (NaCl) was added to the prepared solution and stirred for another 1 h at room temperature. Further, 5 m sodium hydroxide (NaOH) was slowly added into the solution until a pH of 5.3–5.4 was achieved. Later, the solution was shaken vigorously, cooled to room temperature, and kept in a 1 L conical flux. The solution was aged overnight before use. For the fluoride concentration measurement, both sample and TISAB mixture were taken in 1:1 ratio.

## Conflict of Interest

The authors declare no conflict of interest.

## Supporting information

Supporting Information

## Data Availability

The data that support the findings of this study are available on request from the corresponding author.
