# Supplementary material for: Industrial Utilization of Capacitive Deionization Technology for the Removal of Fluoride and Toxic Metal Ions (As3+/5+ and Pb2+)
Source: Glob Chall. 2022 Jan 27;6(4):2100129. doi: 10.1002/gch2.202100129 (PMC8995710; doi:10.1002/gch2.202100129)
Supplement: Supplementary file 1 — Supporting Information [file GCH2-6-2100129-s001.pdf]

Global Challenges
Open Access

Supporting Information

for *Global Challenges,* DOI: 10.1002/gch2.202100129

Industrial Utilization of Capacitive Deionization Technology for the Removal of Fluoride and Toxic Metal Ions ($As^{3+/5+}$ and Pb^{2+})

*Md Rabiul Islam, Soujit Sen Gupta, Sourav Kanti Jana, and Thalappil Pradeep**

Supporting Information

Industrial Utilization of CDI Technology for Removal of Fluoride and Toxic Species ($As^{3+/5+}$ and Pb^{2+})

*Md Rabiul Islam,[1#] Soujit Sen Gupta,[1#] Sourav Kanti Jana[1] and Thalappil Pradeep[1]**

[1]*DST Unit of Nanoscience and Thematic Unit of Excellence, Department of Chemistry, Indian Institute of Technology Madras, Chennai-600036, India*

[#] *These authors are contributed equally to this work*

Corresponding Author

**E-mail: pradeep@iitm.ac.in*

Thalappil Pradeep, DST Unit of Nanoscience (DST UNS) and Thematic Unit of Excellence (TUE), Department of Chemistry, Indian Institute of Technology Madras, Chennai 600036, India.
Tel.: +91-44 2257 4208; Fax: +91-44 2257 0545/0509

Table of Contents

**S. No**	**Description**	**Page**
1	Schematic of CDI experimental set-up	S-3
2	Photograph of CDI experimental set-up	S-4
3	SEM EDS and SEM images of carbon materials	S-5
4	SEM images and SEM EDS of cathode	S-6
5	SEM SEM images and SEM EDS of anode	S-7
6	Raman specra of carbon materials	S-8
7	Cyclic voltammetry (CV) with varying scan rate and specific capacitance vs. scan rate for both electrodes	S-9
8	Cyclic voltammetry (CV) of both cathode and anode in different scan rates in 1 M NaCl and 1 M NaF electrolyte	S-10
9	Nyquist Plot of cathode, anode, and circuit for both electrodes.	S-11
10	CDI performance for removing of F^- with different concentrations (100, 50, and 10 ppm) and the flow rate effect on 10 ppm input F^- solution.	S-12
11	SEM EDS of NaF adsorption after single adsorption on cathode and anode.	S-13
12	Deconvoluted XPS spectra of C 1s of electrodes before and after NaF adsorption.	S-14
13	SEM EDS of cathode after $Pb(NO_3)_2$ adsorption.	S-15
14	SEM EDS of anode after $Pb(NO_3)_2$ adsorption.	S-16
15	SEM EDS of cathode after arsenic adsorption.	S-17
16	SEM EDS of anode after arsenic adsorption.	S-18
17	Fitting vales of an equivalent circuit of both electrodes	S-19

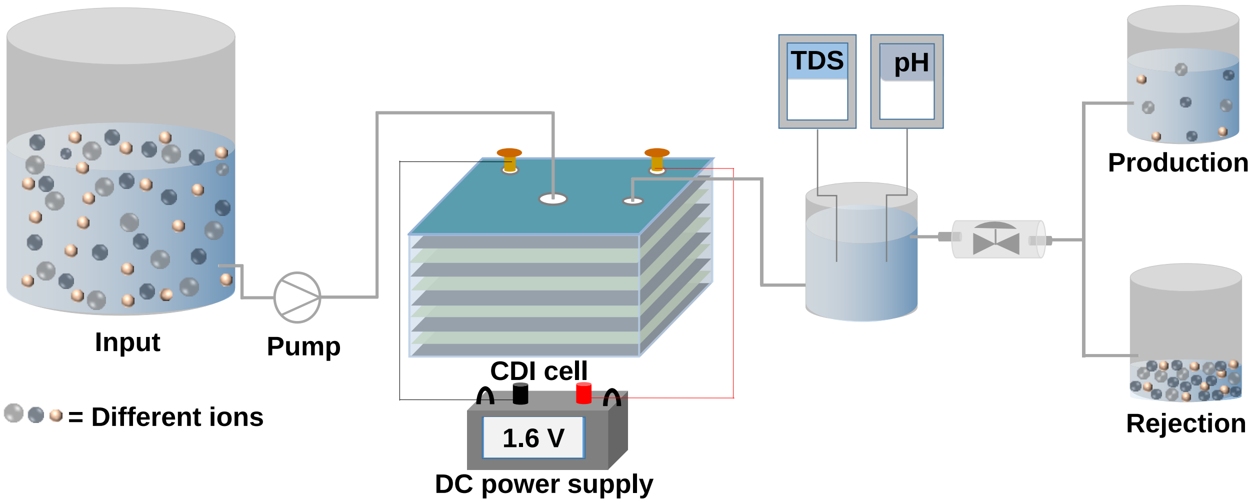

Figure S1. Schematic of CDI experimental set-up.

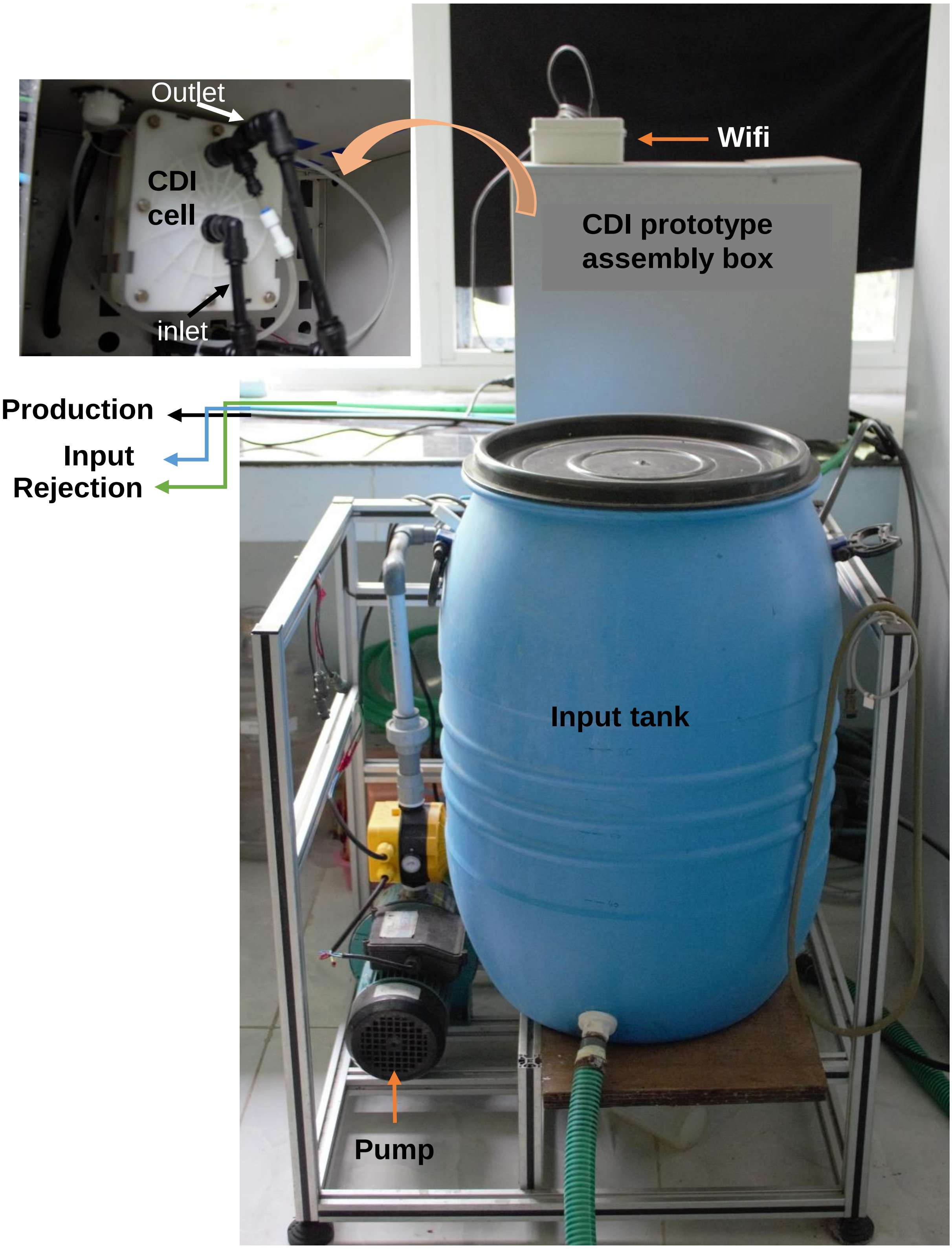

Figure S2. Photograph of the prototype of CDI experimental set-up. CDI box contains CDI cell and controlling electronic boards.

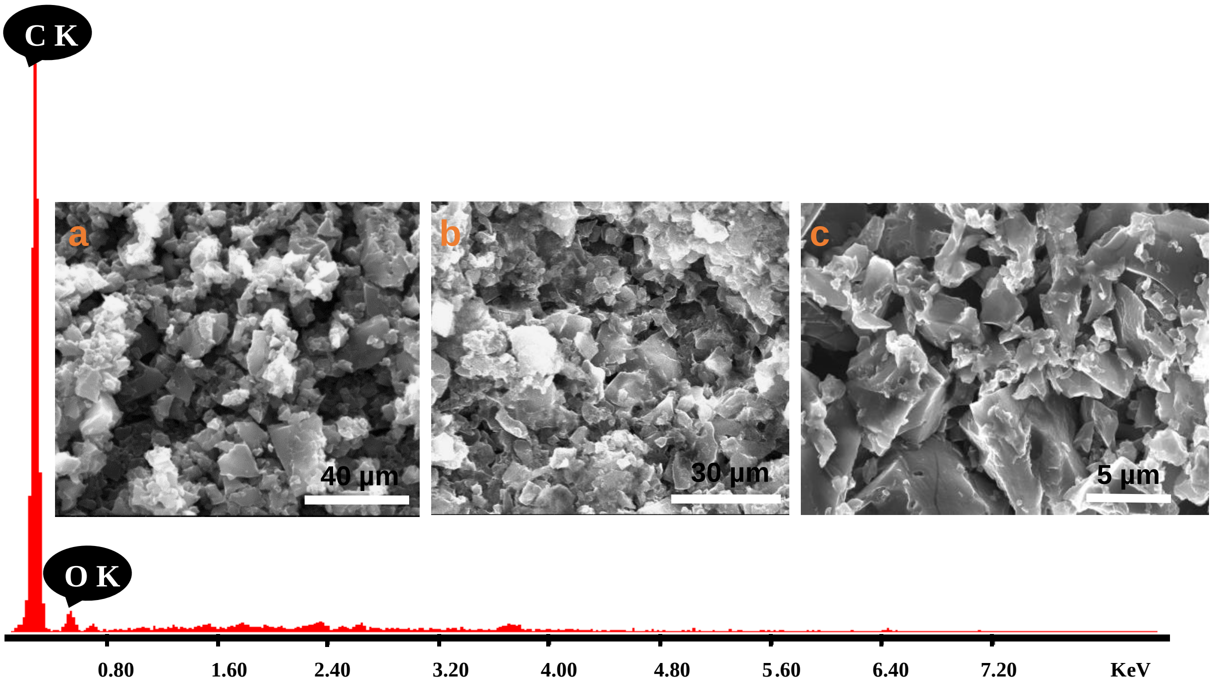

Figure S3. SEM EDS of carbon materials and SEM images are shown in the insets. (Scale bar is 40, 30, 5 μm for a, b, c, respectively)

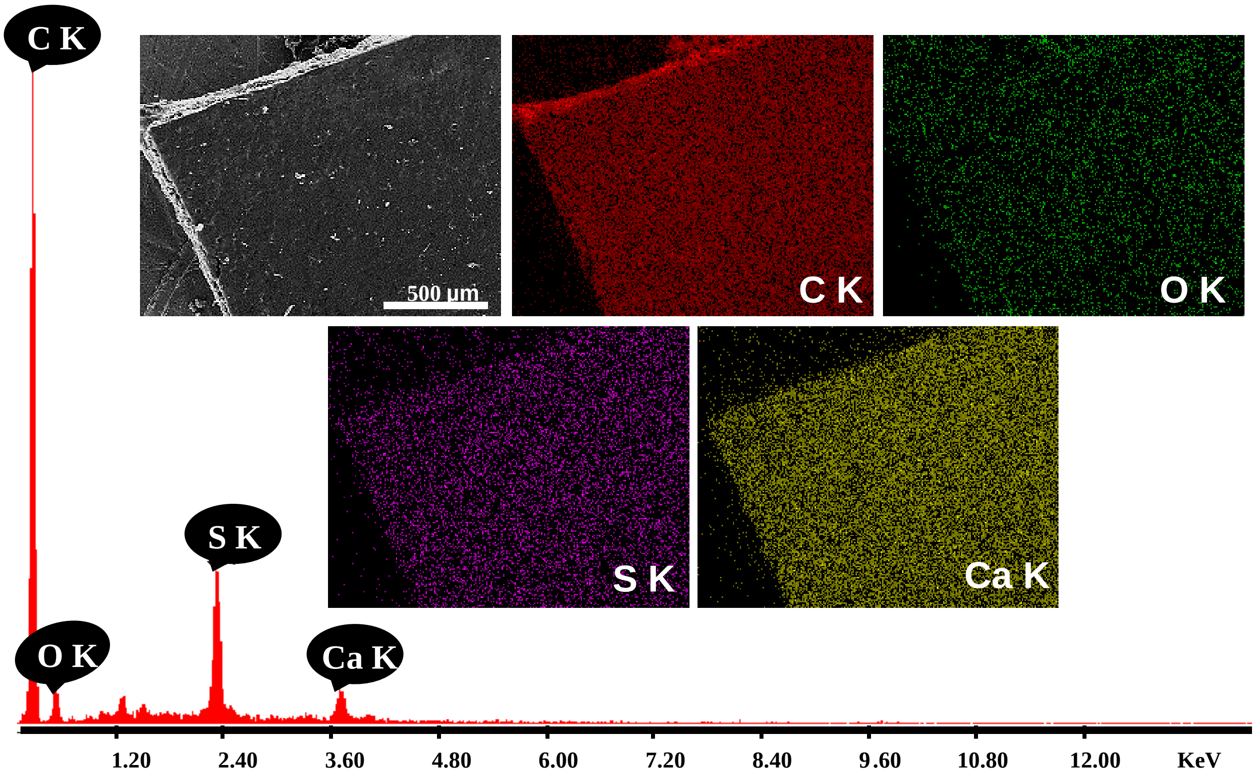

Figure S4. SEM EDS of the cation-exchange resin-coated electrode (cathode). The SEM image and the corresponding elemental mapping images are shown in the insets.

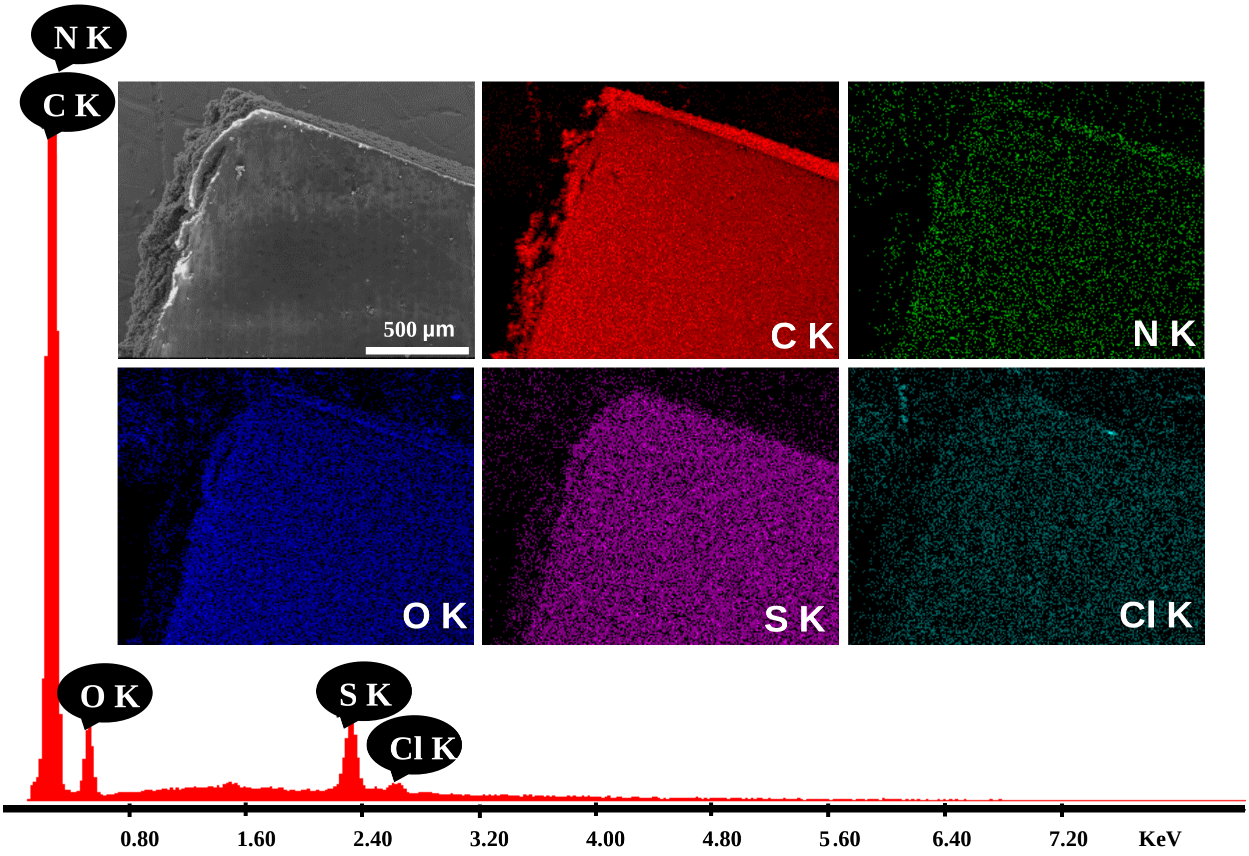

Figure S5. SEM EDS of the anion-exchange resin-coated electrode (anode). The SEM image and the corresponding elemental mapping images are shown in the insets.

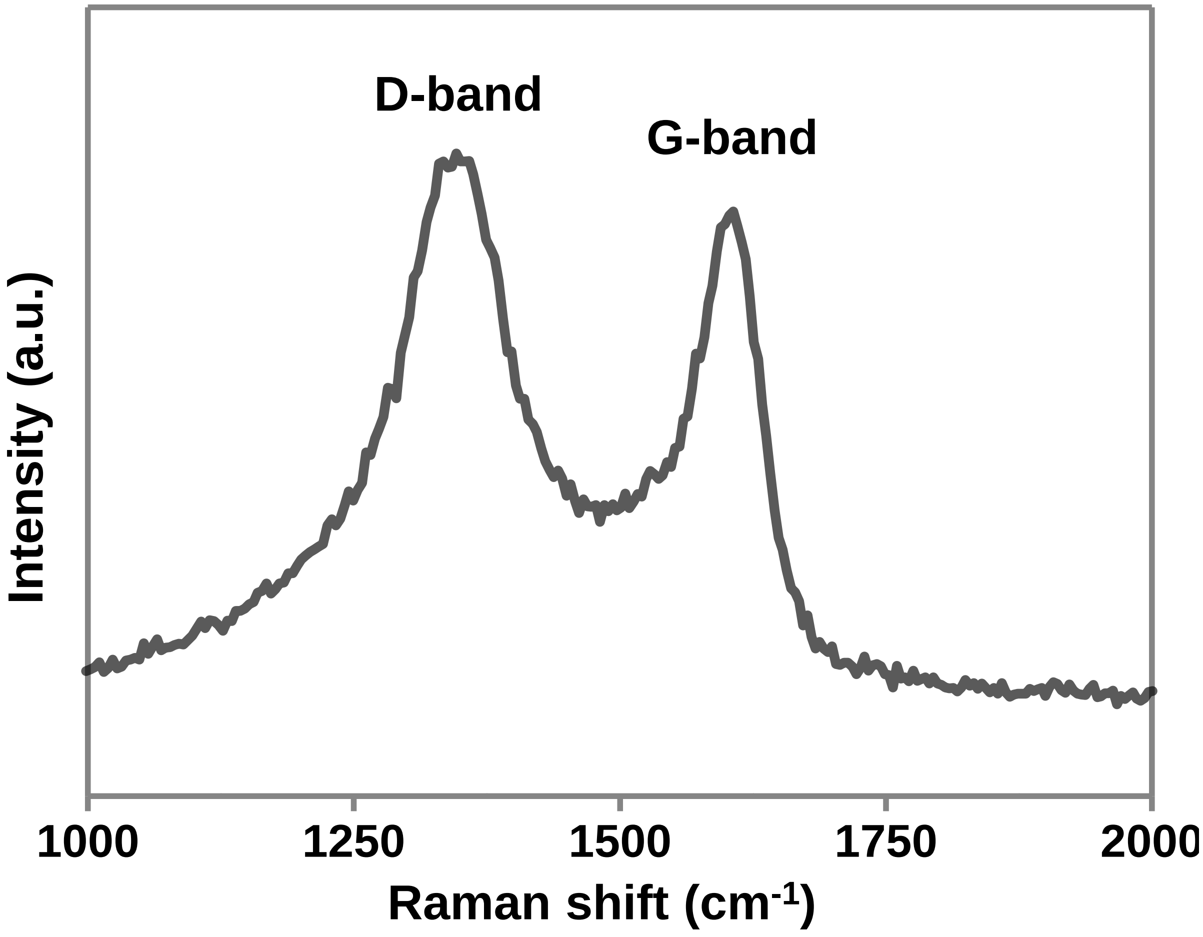

Figure S6. Raman spectrum of carbon materials.

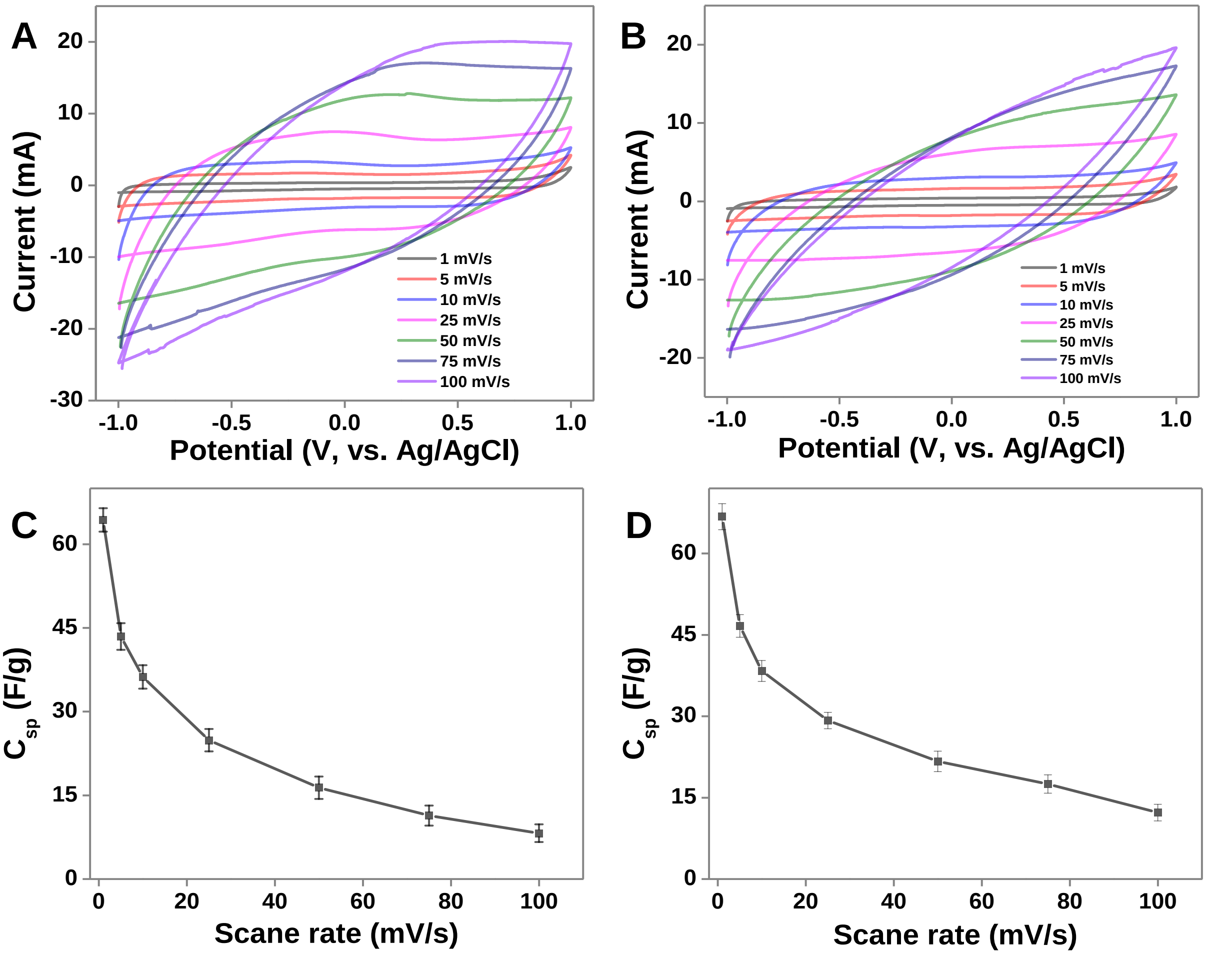

Figure S7. Cyclic voltammetry (CV) with varying scan rates A) cathode and B) anode; C) and D) are specific capacitance vs. scan rates for both cathode and anode materials, respectively. CV potential was varied with respect to Ag/AgCl electrodes using 1 M NaCl solution as an electrolyte.

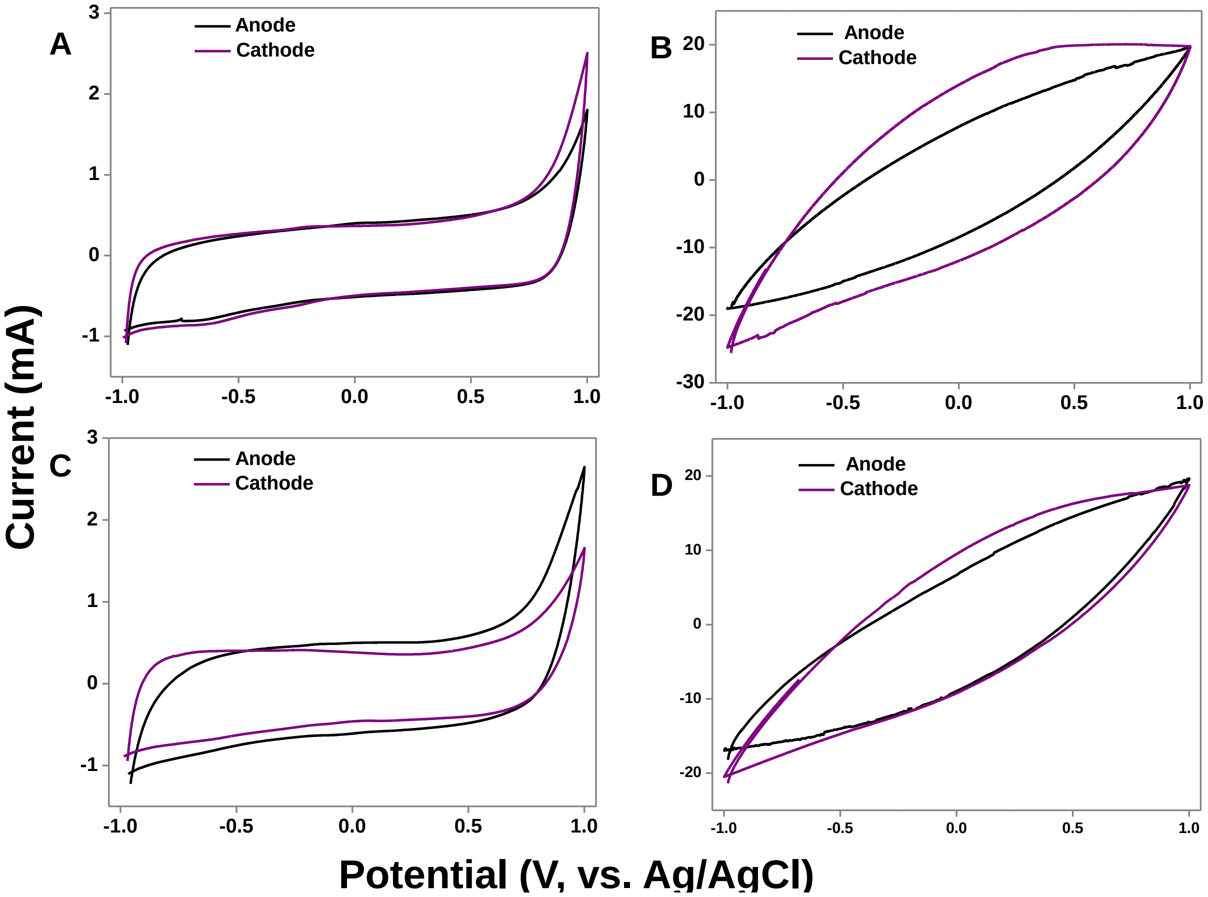

Figure S8. Cyclic voltammetry (CV) of A) cathode and anode both @1 mV/s in 1 M NaCl electrolyte, B) cathode and anode both @100 mV/s) in 1 M NaCl electrolyte, C) cathode and anode both @1 mV/s in 1 M NaF electrolyte and D) cathode and anode both @100 mV/s in 1 M NaF electrolyte.

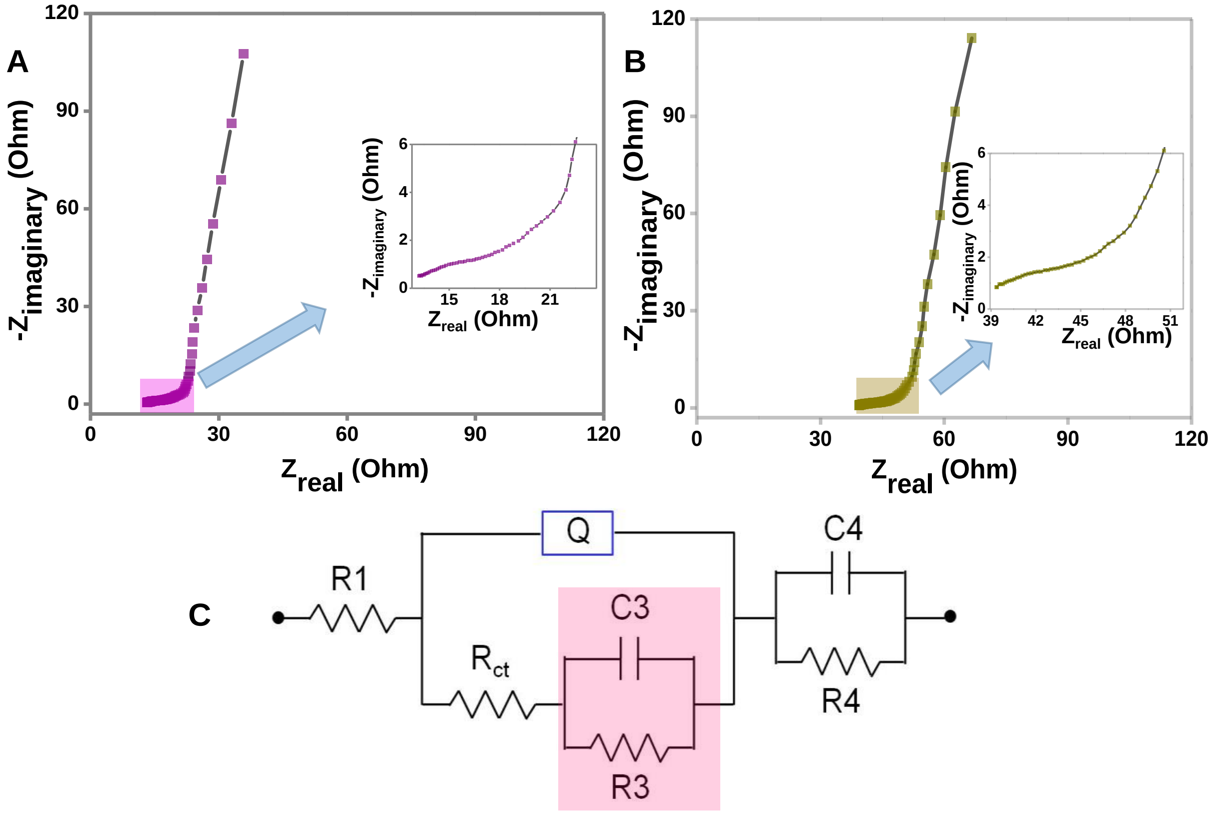

Figure S9. Nyquist Plot of A) cathode (the inset chart shows the magnified high-frequency region), B) anode (the inset chart shows the magnified high-frequency part), C) circuit for cathode and anode.

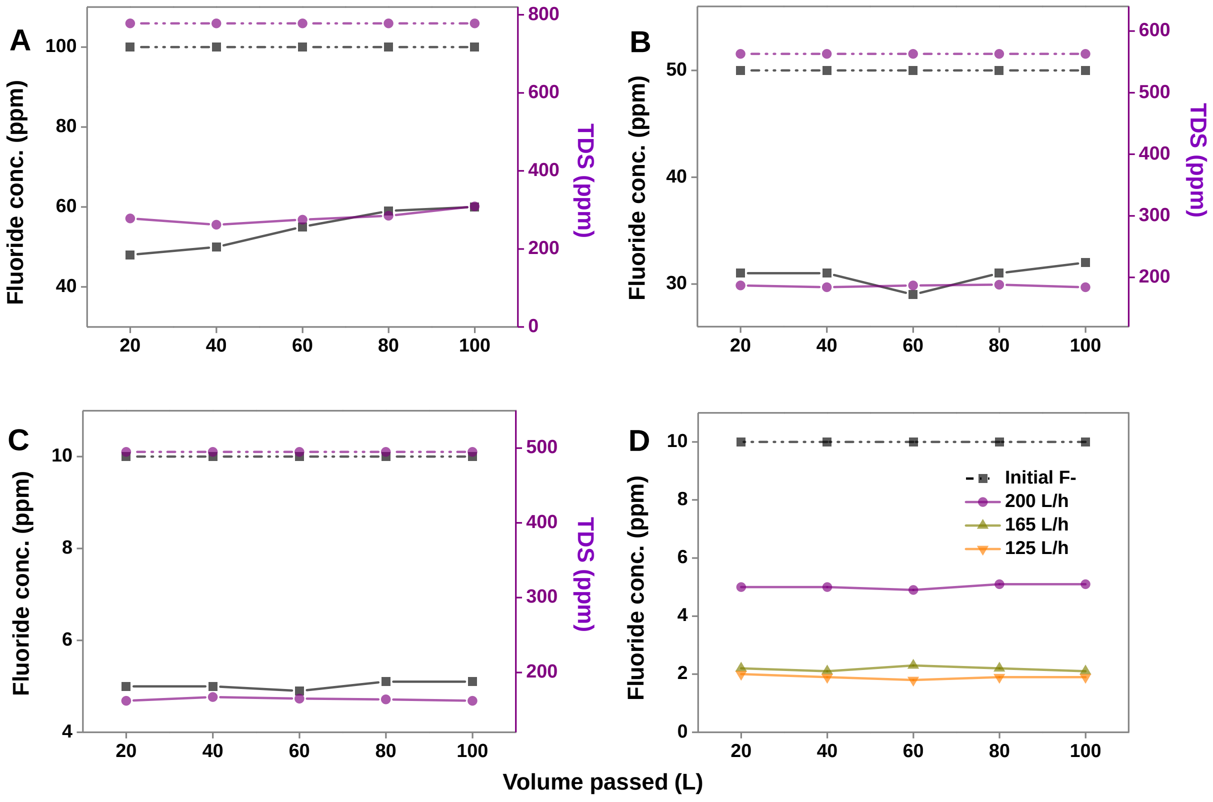

Figure S10. CDI performance for the removal of fluoride ion in tap water with initial concentration A) 100 ppm B) 50 ppm, C) 10 ppm with different TDS, and D) effect of flow rate with 10 ppm input fluoride concentration.

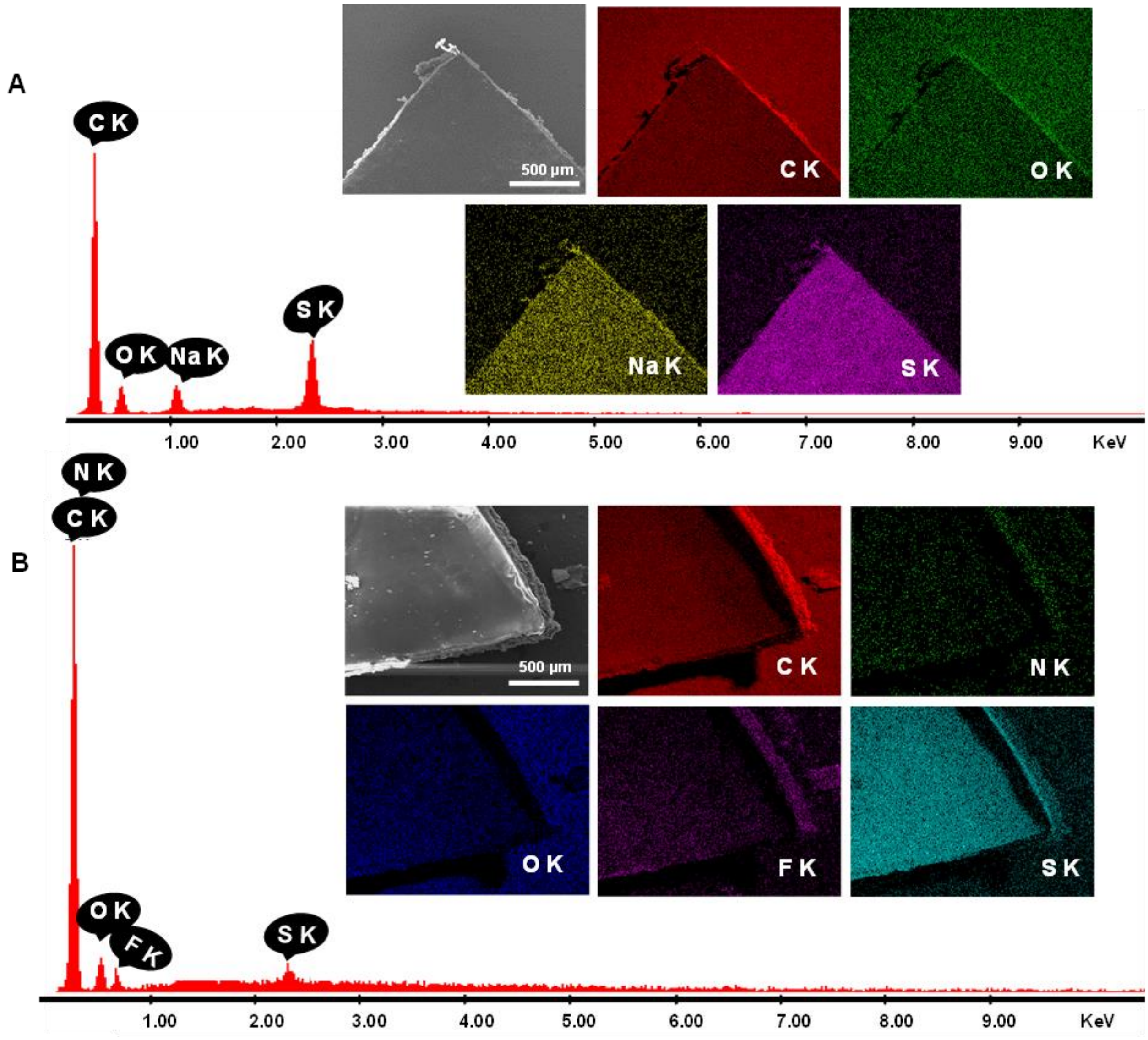

Figure S11. SEM EDS of NaF adsorption after single adsorption on A) cathode and B) anode. The corresponding SEM and elemental mapping images are shown in the inset.

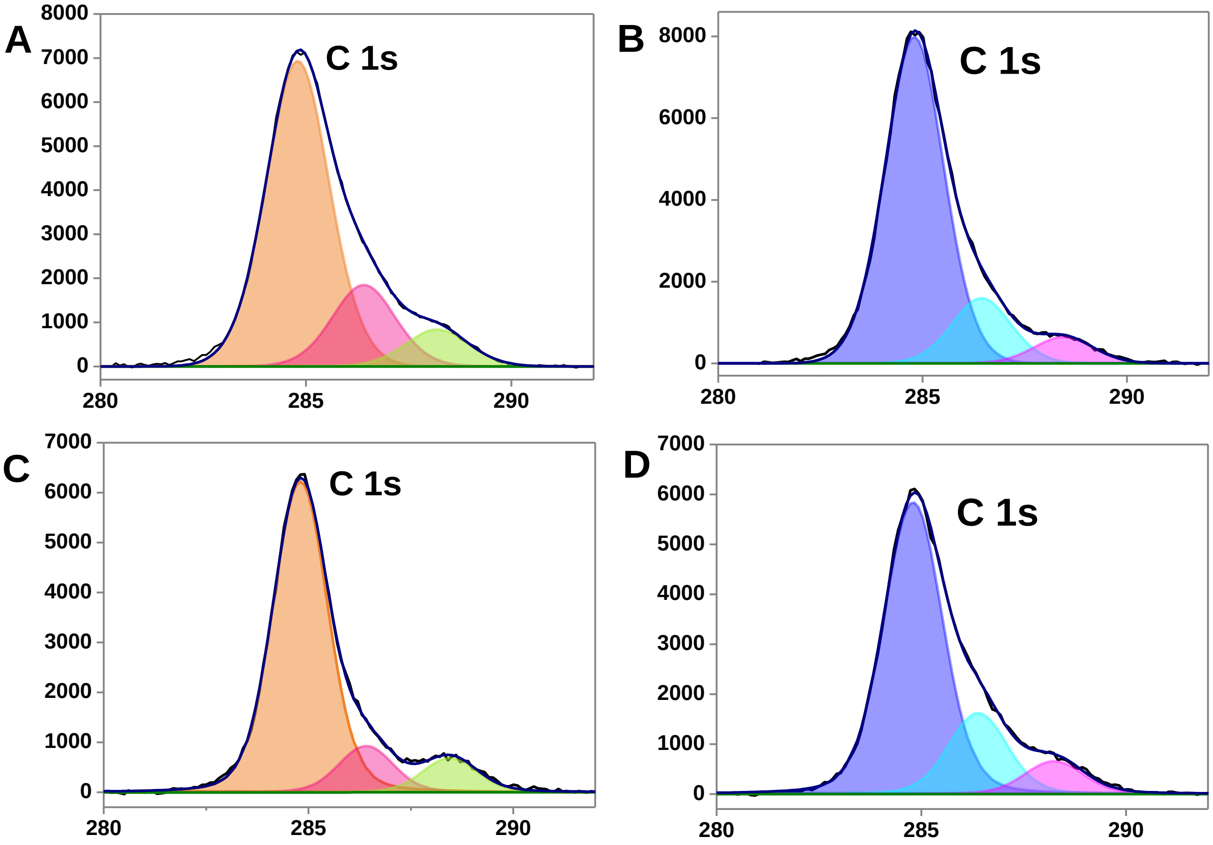

Figure S12. Deconvoluted XPS spectra of C 1s in A) cathode and B) anode before NaF adsorption; C) cathode and D) anode after NaF adsorption.

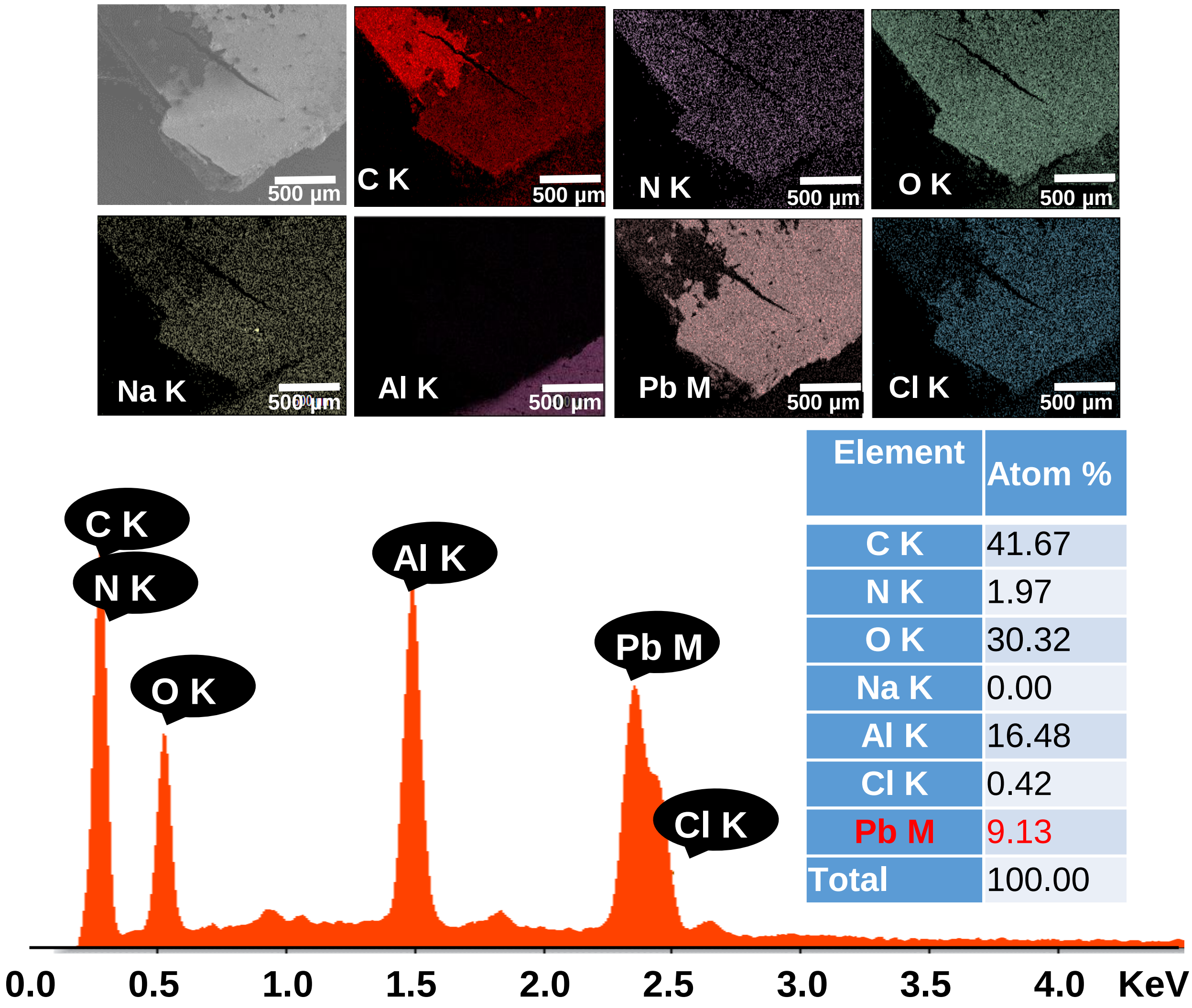

Element	Atom %
C K	41.67
N K	1.97
O K	30.32
Na K	0.00
Al K	16.48
Cl K	0.42
Pb M	9.13
Total	100.00

Figure S13. SEM EDS of $Pb(NO_3)_2$ adsorption after single adsorption on the cathode. The corresponding SEM and elemental mapping images are shown in the inset.

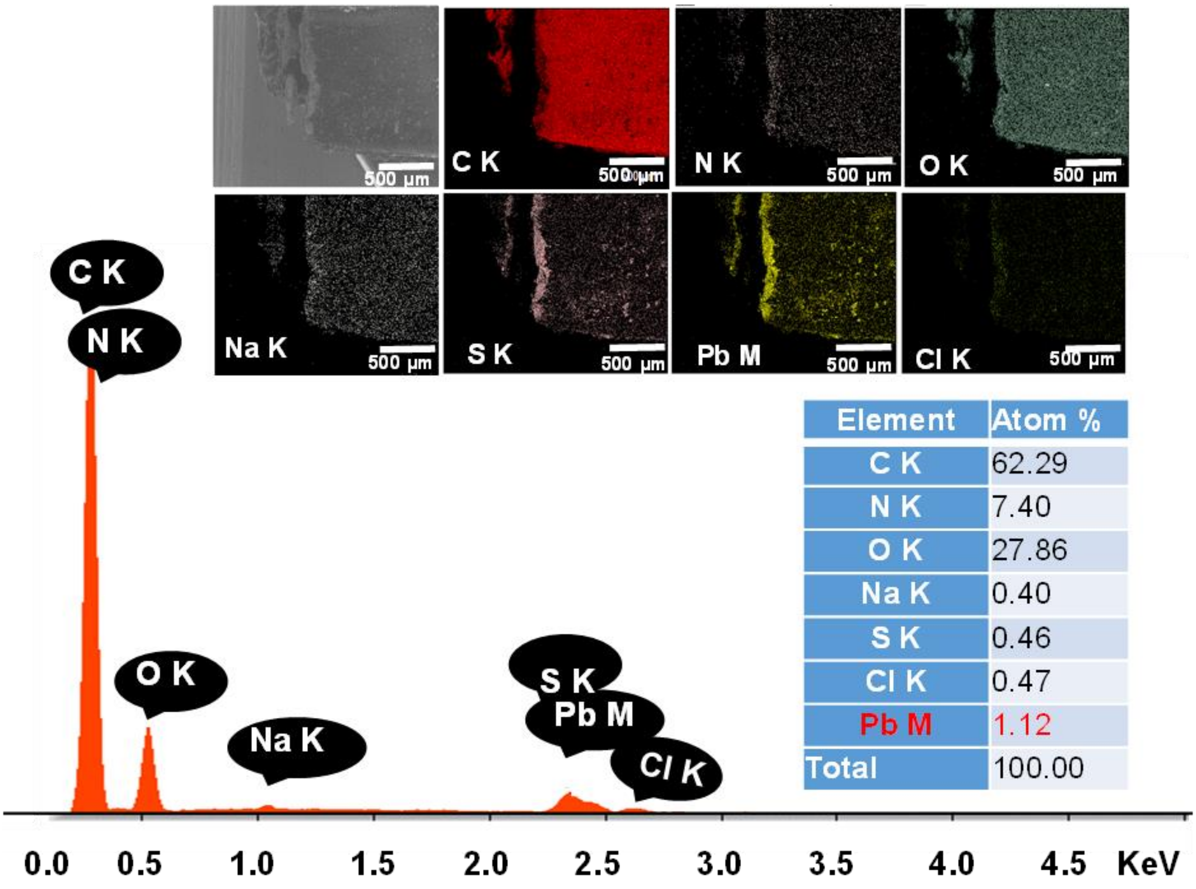

Figure S14. SEM EDS of $Pb(NO_3)_2$ adsorption after single adsorption on the anode. The corresponding SEM and elemental mapping images are shown in the inset.

Element	Atom %
C K	31.40
O K	12.79
F K	1.27
Al K	50.74
S K	0.26
Cl K	0.15
Ca K	2.80
As L	0.59
Total	100.00

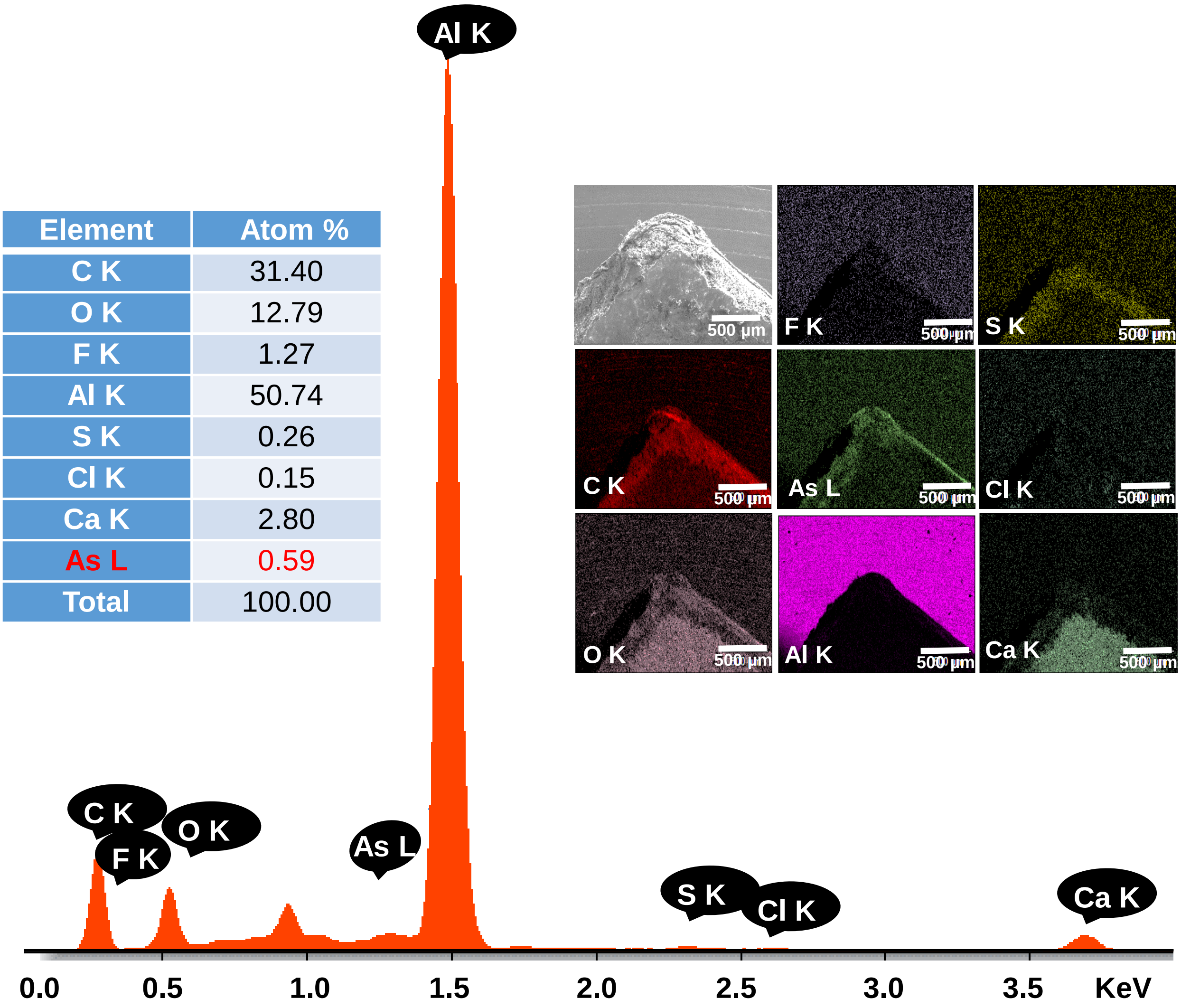

Figure S15. SEM EDS of arsenic adsorption after single adsorption on the cathode. The corresponding SEM and elemental mapping images are shown in the inset.

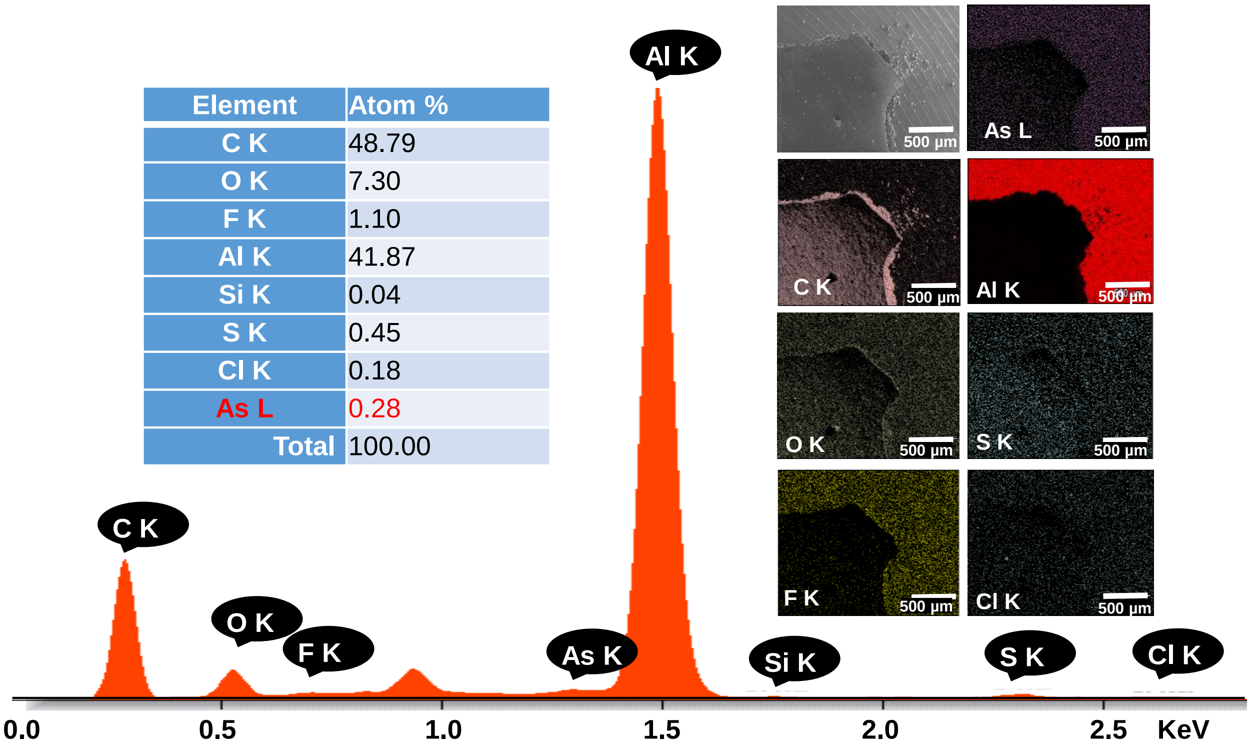

Element	Atom %
C K	48.79
O K	7.30
F K	1.10
Al K	41.87
Si K	0.04
S K	0.45
Cl K	0.18
As L	0.28
Total	100.00

Figure S16. SEM EDS of arsenic adsorption after single adsorption on the anode. The corresponding SEM and elemental mapping images are shown in the inset.

Table S1. Fitting vales of an equivalent circuit of both cathode and anode

Cathode	
R1	12.5 Ohm
Q1	0.06 F.s^(a-1)
a1	0.055
R_{ct}	7 .1 Ohm
C3	16.15e-9 F
R3	1.8 MOhm
C4	0.2905 F
R4	794.3 Ohm

Anode	
R1	38.23 Ohm
Q1	0.04 F.s^(a-1)
a1	0.049
R_{ct}	9 .8 Ohm
C3	16.5e-9 F
R3	5.9 MOhm
C4	0.275 F
R4	831.4 Ohm
